# Efficient hybrid heuristic adopted deep learning framework for diagnosing breast cancer using thermography images

**DOI:** 10.1038/s41598-025-96827-5

**Published:** 2025-04-19

**Authors:** Ahmad Y. A. Bani Ahmad, Jafar A. Alzubi, Manimaran Vasanthan, Suresh Babu Kondaveeti, J. Shreyas, Thella Preethi Priyanka

**Affiliations:** 1 Department of Accounting and Finance, Faculty of Business, Middle East University, Amman, 11831 Jordan; 2 Faculty of Engineering, Al-Balqa Applied University, Salt, 19117 Jordan; 3 Department of Pharmaceutics, SRM College of Pharmacy, Medicine and health sciences, SRM institute of Science and Technology Kattankulathur, Chennai, 603203 Tamilnadu India; 4 Department of Biochemistry, Symbiosis Medical College for Women, Symbiosis International (Deemed University), Pune, 412115 Maharashtra India; 5 Department of Information Technology, Manipal Institute of Technology Bengaluru, Manipal Academy of Higher Education, Manipal, 560064 Karnataka India; 6 Department of Computer Science and Engineering, Saveetha School of Engineering, Saveetha Institute of Medical and Technical Sciences, Thandalam, Chennai, 602105 Tamilnadu India

**Keywords:** Breast cancer classification, Thermogram images, Optimal binary thresholding, Weighted fused feature, Rock hyraxes dandelion algorithm optimization, StackVDRNet, Computational neuroscience, Computational biology and bioinformatics, Health care, Medical research, Biomedical engineering

## Abstract

The most dangerous form of cancer is breast cancer. This disease is life-threatening because of its aggressive nature and high death rates. Therefore, early discovery increases the patient’s survival. Mammography has recently been recommended as diagnosis technique. Mammography, is expensive and exposure the person to radioactivity. Thermography is a less invasive and affordable technique that is becoming increasingly popular. Considering this, a recent deep learning-based breast cancer diagnosis approach is executed by thermography images. Initially, thermography images are chosen from online sources. The collected thermography images are being preprocessed by Contrast Limited Adaptive Histogram Equalization (CLAHE) and contrasting enhancement methods to improve the quality and brightness of the images. Then, the optimal binary thresholding is done to segment the preprocessed images, where optimized the thresholding value using developed Rock Hyraxes Dandelion Algorithm Optimization (RHDAO). A newly implemented deep learning structure StackVRDNet is used for further processing breast cancer diagnosing using thermography images. The segmented images are fed to the StackVRDNet framework, where the Visual Geometry Group (VGG16), Resnet, and DenseNet are employed for constructing this model. The relevant features are extracted usingVGG16, Resnet, and DenseNet, and then obtain stacked weighted feature pool from the extracted features, where the weight optimization is done with the help of RHDAO. The final classification is performed using StackVRDNet, and the diagnosis results are obtained at the final layer of VGG16, Resnet, and DenseNet. A higher scoring method is rated for ensuring final diagnosis results. Here, the parameters present within the VGG16, Resnet, and DenseNet are optimized via the RHDAO to improve the diagnosis results. The simulation outcomes of the developed model achieve 97.05% and 86.86% in terms of accuracy and precision, respectively. The effectiveness of the designed methd is being analyzed via the conventional breast cancer diagnosis models in terms of various performance measures.

## Introduction

The second leading cause of increasing the death rate for women is believed to be breast cancer. The cells shift the breast tissue and separate uncontrollably in breast cells, frequently results tumor or lump. Cancer is divided into two categories: one is benign, another one is malignant. It is also divided into regular and irregular sections. Benign tumors grow relatively slowly and do not spread to other body sections or encroach upon nearby tissues, which can be fatal if not caught in time^1^. Typically, testing can detect breast cancer before any symptoms manifest or after a woman finds a lump. Breast cancer affects more women than any other type of cancer, with an estimated million people receiving the diagnosis worldwide each year. Due to this exponential growth, there is a tremendous need for improving novel prevention of breast cancer methods in their ancient stages^2^. It motivates scientists to strive for novel ways to make rapid and accurate diagnoses, extending patients’ lives.

Regarding prevalence and death, the common illnesses affecting females is breast cancer. There are numerous methods for finding breast cancer. However, dangerous ionizing radiation and the challenges of defining the tumor inside the body are two of this method’s key drawbacks. Infrared thermography has numerous uses in various medical diagnoses^3^. These medical applications cover the diagnosis of cancer and the treatment of diabetes, carotid artery stenosis screening, seasonal influenza detection, eye disease research, and chronic pain management. Because cancer cells generate more heat than normal cells due to their greater metabolic rate, infrared thermography helps to identify developing tumors^4^.

Thermography is a reliable, non-contact, inexpensive, non-invasive technology for screening cancer that can find tumors early on, even in precancerous circumstances^5^. Typical human professionals performed the thermal image interpretation. Previous studies suggested that the logograms resulted from inadequate technical proficiency and experience evaluating such pictures^6^. Gratitude to improvements in thermal sensors, desktop processing capabilities, Artificial intelligence (AI) growth and explosion of deep learning and digital image processing techniques, as well as more affordable and accessible GPU-based. Graphics processing unit-based cloud computing resources^7^. According to some research^8^, thermography can identify early cancer symptoms several years before mammography when done by established guidelines. It enables routine programs of screening in nations with any level of resources and helps minimize diagnostic blunders in breast density and women of any age^9^. The other considerations have prompted research that portrays thermography as an excellent substitute for mammography or, at the very least, as an additional test^10^.

Numerous risk factors raise the likelihood of developing breast cancer^11^. Patients with breast cancer reportedly have risk factor at least one. Thus it’s critical to use the details when calculating the relative risk of contracting the disease. Moreover, this is unable to locate any research that made a diagnosis using the patient’s personal and clinical information from the Document Type Definition Database. It is thought that combining some data with data imaging can significantly increase the capability of diagnosis. Should everyone anticipate that enhance model performance is enhanced by adding patient information^12^ Convolutional Neural Networks (CNNs) is a specific kind of Deep Neural Network (DNN) that use several kinds of neural layers to learn global patterns. CNNs is emerged as strong tool for image processing due to their capacity for automatically extract features^13^. Surprisingly, CNNs is not effectively performed frequently in thermogram-based breast cancer screening given their shown utility^14^. The major goal of this research work is to determine if the model’s ability to detect early breast cancer is enhanced by including lateral pictures with the patient’s personal and medical data^15^. It is thoroughly researched and evaluated how each sort of patient information affects the models’ capacity to make diagnoses^16,17^. The following is a list of the main goals of the suggested categorization model for breast cancer.

- Implementing this novel breast cancer diagnosis model with thermogram images and using a hybrid heuristic-aided stack deep learning model helps diagnostic centers and clinical institutes detect the disease rapidly.
- To segment the abnormality in pre-processed images by adopting the optimal binary thresholding, where the threshold value is optimized using RHDAO. It leads to improving the performance classification.
- To extract the features using the VGG16, ResNet, and DenseNet, this is then used to obtain the stacked fused features with the help of weight optimization done by RHDAO. It assists in easily finding the essential features to enhance performance.
- Feed the fused features into StackVDRNet, which includes VGG16, ResNet, and DenseNet. To acquire the optimal results, the parameters such as VGG16 in epochs, ResNet in the activation function and DenseNet in the hidden neuron are tuned by RHDAO to maximize the accuracy.
- Examine the proposed model with divergent measures and evaluate it with traditional classifiers and optimization algorithms.

Part II, which covers current breast cancer categorization literature, comes after the introduction. Part III elucidates the dataset and architecture and describes the novel diagnosis model and part IV describes the hybrid heuristic algorithm and optimal binary thresholding segmentation. The proposed StackVDRNet is shown in Part V. Part VI elucidates the experiment’s findings. In part VII, the paper is finally finished.

## Systematic review

### Existing works

In 2025, Richa et al.^18^ have implemented Deep Learning Framework (DLF) leveraging with various phases with the help of Artificial Neural Networks (ANN) to categorize breast cancer. Moreover, the experimental findings of the model have demonstrated to increase the scalable performance considering diverse performance measures.

In 2019, Dina et al.^19^ have implemented a novel deep learning model to classify the breast cancer. Initially, Computer Aided Detection (CAD) was employed by classifying malignant and benign tumors. For extracting the relevant features, the DCNN model was applied. Moreover, the developed model could be train massive amount of data to enhance accuracy. Extensive experiments have been conducted for maximizing efficiency of the model with baseline techniques.

In 2021, Mukhmetov et al.^20^ have proposed combining thermography with numerical analysis to detect breast tumors inside the breast utilizing patients’ unique data, such as specific patterns in temperature and breast form. Previous research relied on idealistic, semi-spherical breast models without implementation verification and patient-specific data. Accurate 3D breast geometry was used in this study’s numerical model, which was further validated in tests by creating a breast using moulding and 3D printing. This improved the precision and dependability of Computer-Aided Diagnosis (CAD)of breast cancer. Breast shape was found to be important in determining the temperature field and recognizing the breast tumor in the combined computational and experimental research. As a result, it was determined that tumor printing and breast geometry were related.

In 2020, Zarif et al.^21^ have developed the hybrid CNN + EfficientNetV2B3 model for identifying the positive as well as negative tissue with the help of Whole Slide Images (WSIs). Here, the pre-trained model has been employed to diagnose breast cancer. Training the model with existing conventional techniques has proved the accurate outcomes, especially with diverse evaluation metrics.

In 2023, Khalid et al.^22^. have suggested the effective deep learning model for identifying the breast cancer. Here, the work has suggested the feature extraction mechanism to extract the low-level features in an effective manner. For the implementation process, the developed model has outperformed with diverse classifier models to validate the outcome.

In 2021, Houssei et al.^23^. have proposed an innovative, efficient during the initialization phase of the search space. “Opposition-Based Learning (OBL)” was utilized to broaden the population diversity of the Chimp Optimization Algorithm (ChOA), and the Lévy Flight was demonstrated. In addition, the multilayer thresholding Image segmentation problem was approached using the ChOA. The proposed method was assessed during the optimization phase utilizing the Kapur and Otsu methods on data from the Mastology Research using the Infrared database. According to outcomes, the Improved ChOA (IChOA) obtained accurate outcomes utilizing the evaluation matrices “Peak Signal to Noise Ratio (PSNR), Structured Similarity Index Method (SSIM), and Feature Similarity Index Method (FSIM)” adopting the fitness concept. In the end, IChOA outperformed rival approaches for ensuring better robustness with several positive and negative cases.

In 2024, Rahman et al.^24^ have suggested the complex DCNN model integrated with other deep learning models like U-Net and YOLO for identifying breast cancer. By leveraging this technique, the automatic identification of breast cancer was highly performed using mammography images. In order to access the model, the thorough experimental analysis was validated for diagnosing breast cancer during the screening process. The performance evaluation of the method was conducted with extensive experiments with diverse performance measures.

In 2024, Naz et al.^25^. have implemented a novel medical diagnostic system utilizing with IoT system. Here, the developed model has outperformed with the presence of tumors. Adjusting the hyperparameter was done by considering the CNN modelfor enhancing the accuracy. The experimental findings of the technique revealed to enhance the patient outcomes using IoT technology.

In 2023, Wang et al.^26^ have recommended an adaptive enhancement technique by adopting with the help of virtual exposure strategy. The experimentation was conducted with diverse conventional approaches among various benchmark datasets to enhance the image quality. In 2025, Wang et al.^27^ have formulated the infrared Fluorescent Probe (FJ-AChE) for high selectivity, sensitive detection of Acetylcholinesterase. In 2024, Liu et al.^28^ have investigated the different stages of anesthesia using Near-Infrared Spectroscopy (NIRS) signals with machine learning. In 2023, Cheng et al.^29^ have investigated the Surface Enhanced Raman spectroscopy (SERS) model was designed for discovering the biomarkers of breast cancer. Throughout the empirical findings, the performance of the model has attained 100% by focusing the accuracy, specificity and sensitivity. In 2023, Zheng et al.^30^ have designed a fast and low-cost diagnosis method by utilizing deep learning as well as serum Raman spectroscopy techniques for detecting breast cancer.

### Problem statement

A robust data storage system is needed for the breast cancer diagnosis model, but it cannot guarantee accurate findings for people with dense breasts or those undergoing surgical procedures. Sometimes, they suffer from misdiagnosis results during a breast cancer diagnosis. It affects the technical performance, quality of practices, and patient-centered work. Many deep-learning techniques have been improved for diagnosing breast cancer using thermography images; some are listed in Table 1.DLF^18^ layers are sparsely connected rather than fully connected. So, it is easy to train. Also, it is simple to understand and implement. However, it gives low-resolution results during segmentation and increases false negative rates.

Deep learning^19^ makes it easy to maintain memory saving and enhance diagnosis performance. Also, gives the efficient quality of segmented images for improving the breast diagnosis results. Yet, the involvement of ionizing radiation affects the early visualization of dense tissues and leads to misdiagnosis results during the detection. TL^20^ Women without obvious cancer symptoms can have their cancer risk efficiently evaluated using screening thermogram images. Additionally, efficiently identifies cancer at an early stage. But, it is an unpleasant, disturbing, and painful procedure, and also Computational time is expensive. CNN + EfficientNetV2B3^21^ avoids the discomfort of the patients during the breast cancer diagnosis. Also, it is very efficient in high-dimensional search solution. Yet, it struggles to detect breast oncology in females with thick breasts, and sometimes target classes overlap. Hence, it decreases the performance in terms of accuracy. Efficient deep learning^22^ supports real-time scanning, contactless for breast cancer diagnosis. Also, it provides transformation shape-dependent information and stability information extraction from acoustic images to improve the model’s capacity. Yet, it provides radiation for the diagnosis process and affects the patients. Suffering from the diagnosis model radiation is also not good for extraction. OBL^23^ increases life expectancy and decreases the associated mortality rate.

Additionally, it produces outcomes that are accurate, high-quality, and consistent. But, sometimes, the test results may be normal even though they have cancer, and it is also ionizing, so difficult to diagnose breast cancer. DCNN^24^ increases the robustness and is more reliable for breast tumors. Also, diagnostic performances are improved by adding quality information. Diagnosis using thermography images with advanced deep learning techniques. However, the cost is rather exorbitant because it is intrusive and risky, and periodic mammography screening programs are not yet available in some developing nations. CNN^25^ reduces the proportion of women receiving a late-stage cancer diagnosis.

Additionally, it helps to lower mortality. However, it has low diagnostic accuracy and makes it challenging for radiologists to make the right diagnosis. These difficulties encourage us to create effective breast cancer treatments.

**Table 1:** Benefits and challenges of traditional breast cancer detection.

Author [citation]	Methodology	Benefits	Limitations	Result quantification
Richa et al.^18^	DLF-ANN	• Layers are sparsely connected rather than fully connected. So, it is easy to train. • It is simple to understand and implement.	• It gives low-resolution results during segmentation. • It increases the false negative rates.	By validating this model, the simulation findings have demonstrated 94.93% and 89.21% regarding accuracy and recall measure.
Dina et al.^19^	Deep learning	• It is easy to maintain memory saving and enhance diagnosis performance. • It gives the efficient quality of segmented images for improving the breast diagnosis results.	• The involvement of ionizing radiation affects the early visualization of dense tissues. • It leads to misdiagnosis results during the detection.	It ensures high classification accuracy shows 87.2%.
Mukhmetovet al.^20^	TL	• Screening thermogram images are a useful tool for assessing cancer risk in females without obvious symptoms.	• It is disturbing, painful and unpleasant, procedure. • Computational time is expensive.	–
Zarif et al.^21^	CNN + EfficientNetV2B3	• It avoids the discomfort of the patients during a breast cancer diagnosis. • It is very effective in high-dimensional spaces.	• It struggles to diagonalizable breast cancer in women with dense breasts. • Sometimes target classes overlap. Hence, it decreases the accuracy.	Comparing with diverse performance measures, the accuracy, recall and F1-score of the model has achieved 96.3%, 86.4% and 89.7%, respectively.
Khalid et al.^22^	Efficient deep learning model	• It offers transformation shape-dependent information and stability information extraction from acoustic images to improve the model’s capacity. • It facilitates real-time scanning, contactless for the diagnosis of breast cancer.	• It provides radiation for the diagnosis process, and it affects the patients. Suffering from the diagnosis model radiation. • It is not good for extraction.	–
Houssei et al.^23^	OBL	• It increases life expectancy and decreases the associated mortality rate. • It achieves valuable and accurate results regarding accuracy, quality, and consistency.	• Sometimes the test results may be normal even though they have cancer. • It is ionizing, so difficult to diagnose breast cancer.	In this model, the performance shows effective and reliable outcomes concerning PSNR, SSIM and FSIM respectively.
Rahman et al.^24^	DCNN	• It increases the robustness, and it is more reliable for breast tumors. • Diagnostic performances are improved by adding quality information.	• It is really expensive. Therefore, some developing nations do not have the means to implement a screening program that includes routine mammograms. • It is invasive and unsafe.	Accuracy rate of 93.0% is achieved in this model.
Naz et al.^25^	CNN	• It decreases the number of females to diagnose with late-stage cancer. • It is used to reduce mortality.	• It gives low diagnostic accuracy. • It is still difficult for radiologists to take proper decisions about the diagnosis.	Accuracy rate of 95% is achieved in this model.

## Thermography images and detection of breast cancer using deep hybrid learning

### Thermography datasets for breast cancer

**Fig. 1:**
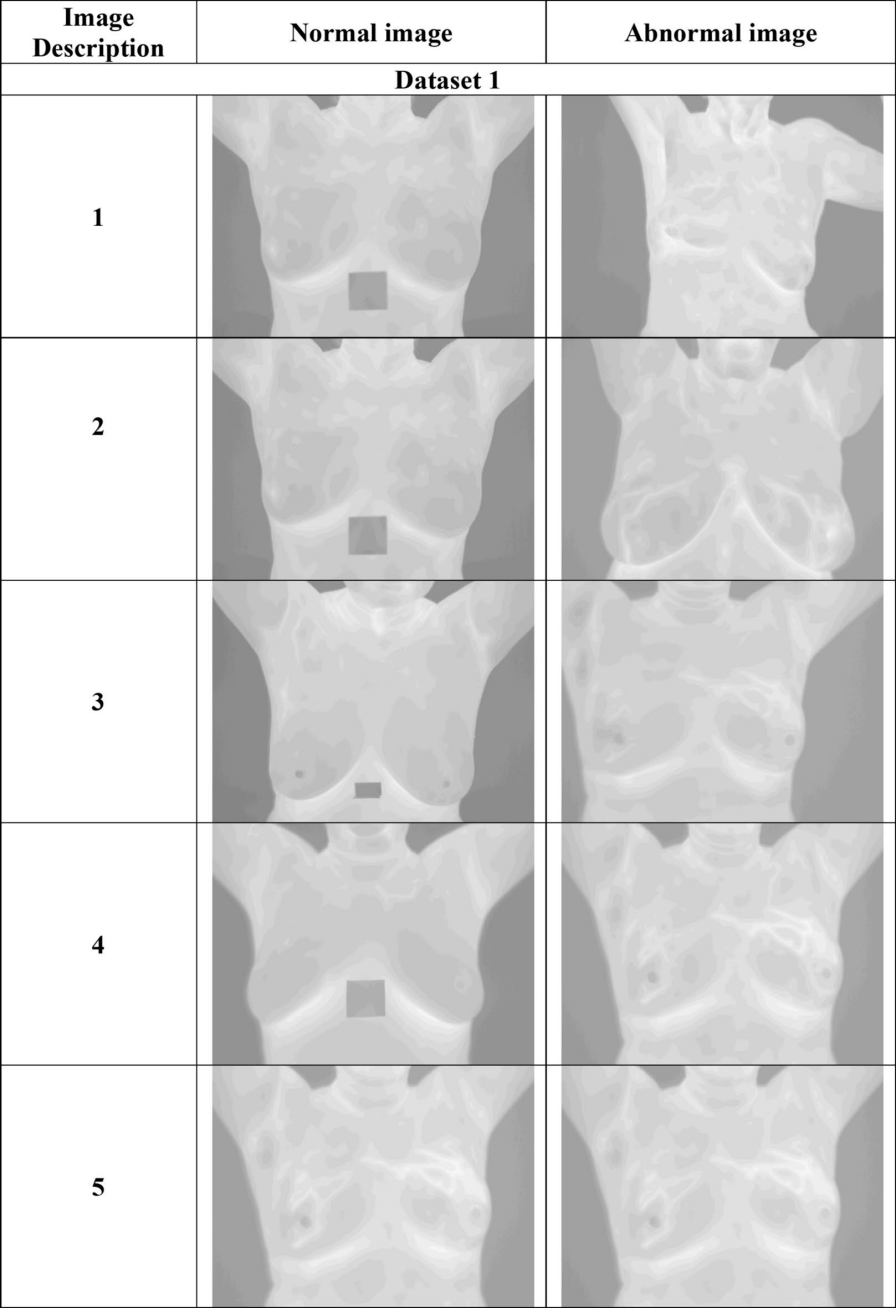
Sample images of datasets for normal and abnormal breast cancer.

The Link is “https://www.kaggle.com/datasets/asdeepak/ hermal-images-for-breast-cancer-diagnosis-dmrir”: “Access Date: 2023-02-02. Thermal Images for Breast Cancer Diagnosis DMR-IR”, thermal pictures obtained using the dynamic protocol were utilized to document the returning the process of patient’s body the environment with equilibrium. After cooling the breasts with an air stream, 20 sequential photographs were acquired at intervals of 15 s. The photos can be found on the website http://visual.ic.uff.br/dmi in the Database for Research Mastology with Infrared Image-DMR-IR. Additionally, the approach had perfect accuracy with them. In conclusion, a sequential thermography-based Computer-Aided Diagnostic (CAD) system approach is being suggested. The total image from this dataset is counted as 1800 and the size of the dataset is 2.12 GB. The collected images are represented by${T_i}$where ${T_i}$the total images collected in the data set. Some abnormal and normal images of breast cancer images are given below. Figure 1 shows both sample images for normal and abnormal breast cancer classification.

### Developed framework for breast cancer detection

Breast cancer is the most usual oncology in women. One in eight women develop this malignancy over their lifetime. Although it is uncommon, it can affect both men and women. A malignant tumor that originates from breast cells is called breast cancer. Some of these risk factors lead to the development of malignant cells. Cancer is screened for before a person exhibits any symptoms. Early cancer detection can be beneficial. The likelihood of recovery is higher when cancer is discovered sooner. Due to restrictions in imaging methodology, it can be difficult to detect abnormalities with traditional breast thermography. Rotational thermography is a cutting-edge method created to overcome traditional thermography’s drawbacks in the breast^31,32^. Traditional breast thermography requires the patient to sit at a certain distance in front of the camera. Due to regular breast drooping, the lower posterior breast areas are not completely visible in these pictures. Malignancies in these regions are so commonly overlooked^33,34^. Thermogram images have less noise and always have distinctive illumination as a result. Classifying breast cancer images naturally falls within fine-grained visual object classification^35^. The first is a forward problem that involves calculating the scattered field using known microwave excitations and distributions of dielectric properties.

**Fig. 2:**
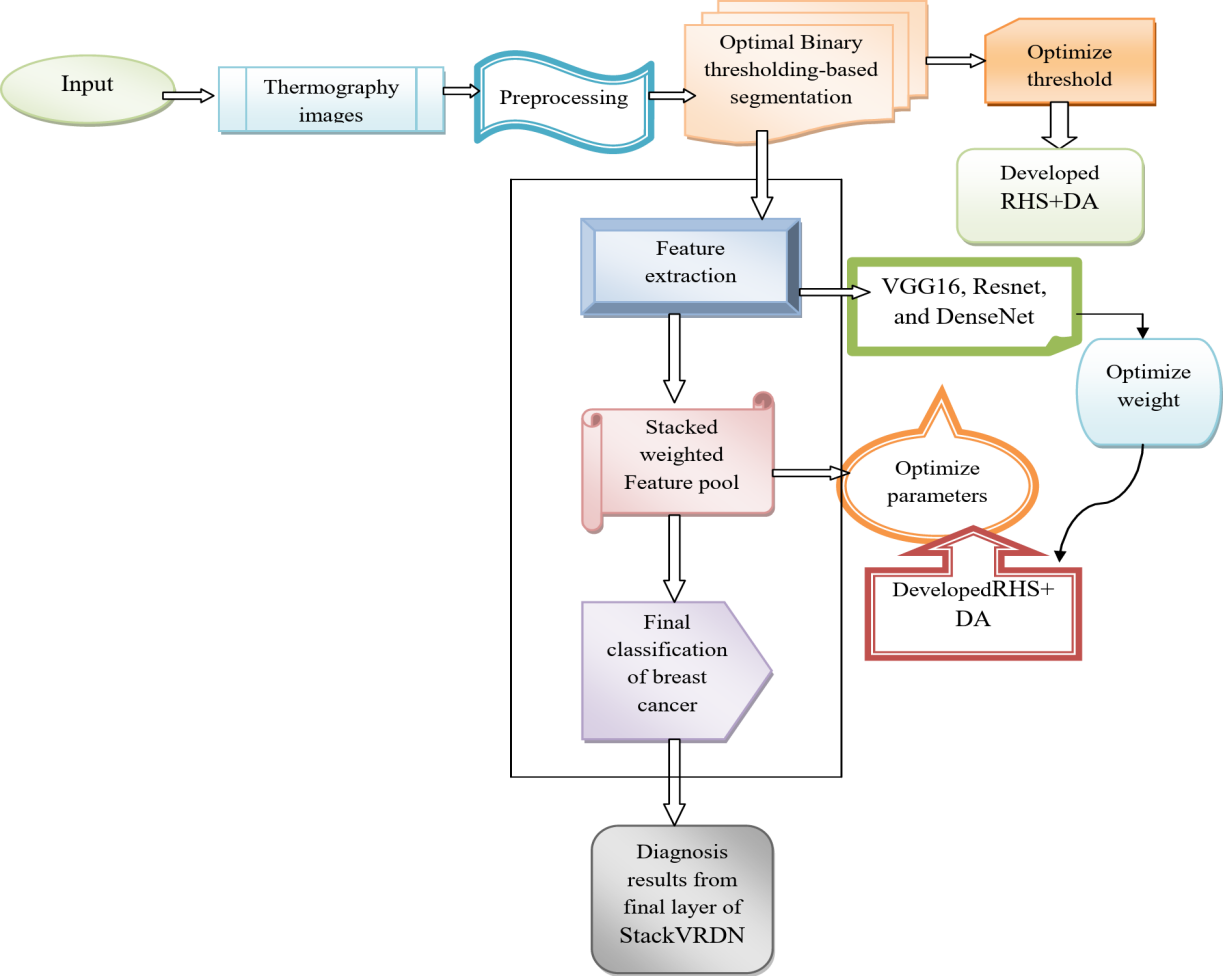
Architectural diagram of new breast cancer classification using thermogram images.

Non-invasive, cost-effective method to lower clinical errors in the diagnostic process is computer-aided diagnosis^36^. It is a supporting tool for practitioners to employ throughout their daily tasks using computer algorithms. Automated classification algorithms are forecasting models that help identify breast cancer^37^. These classification algorithms’ guiding premise is the identification of characteristics that distinguish various classes from the training dataset^38,39^. The SVM has different kernel functions, including, linear, Sugeno Fuzzy, polynomial, Radial Basis Function (RBF), Decision Tree (DT), and k-Nearest, the most widely used classifier. A promising screening method that can help diagnose the disease is using infrared imaging to diagnose breast cancer^40^. It is a painless method that can detect changes in thick breasts, has no harmful radiation, and can see early alterations, but it improves accuracy and efficiency. Figure 2 shows the Architectural diagram of novel breast cancer classification using thermogram images.

A novel deep learning-based breast cancer diagnostic method is adopted using the thermography images. The thermography images needed to diagnose breast cancer are initially gathered from online resources. To develop the brightness of the images and quality of images, the gathered thermography images are preprocessed using CLAHE and Histogram. The preprocessed images are then segmented using the best binary thresholding, where the thresholding value is optimized using a newly created RHDAO. The thermography images are further processed to detect this oncology with the use of therecently built deep learning structure StackVRDNet. Segmented images are given into the StackVRDNet model that is constructed by VGG16, Resnet, and DenseNet. With VGG16, Resnet, and DenseNet, the pertinent features are extracted. From there, a stacked weighted feature pool is created, and the weight is optimized using the same RHDAO. The diagnosis results are obtained at the last layer of VGG16, Resnet, and DenseNet, and the higher-ranking approach is used to obtain the final diagnosis findings. The final classification is carried out using StackVRDNet. Here, the VGG16, Resnet, and DenseNet parameters are improved using the RHDAOto maximize diagnosis accuracy. In parameter of some performance indicators, the effectiveness of the created model will be evaluated using the traditional breast cancer diagnosis models. These features achieve more efficiency and enhance the model.

### Thermography image pre-processing

Preprocessing is the first step that must be completed before beginning any digital image processing operation to improve the best possible results. Preprocessing is the first phase of the processing of digital images. The major objective of pre-processing is enhancing image quality by reducing noisy pixels. Here, the CLAHE and Histogram Equalization are performed to enhance the image pre-processing and ensure better quality images. In thermography images, the CLAHE model is leveraged to automatically image contrast by reducing the presence of noise level. Furthermore, the CLAHE model provides the clip limit parameter for controlling the contrast enhancement and preventing excessive amplification of noise.To enhance image contrast, the histogram equalization model is adopted in the pre-processing process. Generally, the histogram equalization model effectively estimates the pixel intensities in thermogram images with the help of Cumulative Distribution Function (CDF). Then, each pixel value gets mapped to a new value and effectively spread the intensities across the whole range. Distributing the intensity value evenly in the images can provide better visualization outcomes and effectively increase the image contrast. In histogram equalization model, the selection of tile size plays a essential role in the thermography images to get the desired outcome. Too small and too large tile size can leadsto excessive noise amplification and not effective to capture fine details. So, the clipping limit parameter in this model is adjusted by reducing the noise level in the thermography images that significantly enhance the contrast of the images. Here, two common techniques are employed that are illustrated below.

CLAHE^41^: Here, the gathered raw image ${T_i}$ is taken as input on a histogram basis; CLAHE has been widely utilized for picture enhancement. More precisely, the algorithm is founded on the presumption that an image maintains consistency. One particular grayscale mapping is used to improve all of those sections of the image. However, the grayscale distribution varies, indicating that a technique for equally distributing grey in images must exist. The discovery of each pixel subject to its neighbor population of grayscale is supported by the adaptive histogram equalization approach. CLAHE accomplishes this technically by establishing a threshold. The image won’t be overly improved after this processing, and the noise amplification issue can be lessened.

There are two steps in the CLAHE process. First, the image must be split into several equal-sized, non-overlapping areas. The clip limits for histogram clipping are then determined. The height of each histogram is then redistributed so that it does not exceed the clip limit. The clip limit is discovered, *β* which may be expressed in Eq. (1).

1 $$\beta =\frac{{AB}}{N}(1+\frac{\alpha }{{100}}({L_{\hbox{max} }} - 1)$$

From the above equation *β* is the limit of the clip and *A×B* is the grayscales of numbers. α Are the clip’s factor and the slope maximally allowable.

From this Eq. (1) $\alpha =0$, then the limit of clip =$\frac{{AB}}{N}$.Set four X-ray images in$L{}_{{\hbox{max} }}$. When given${T_i}$as images at the output given the input as pre-processed images. The resultant image of the CLAHE model is obtained, and it is denoted by ${T_i}$.

Histogram Equalization^42^: Here, the acts input HE as a widely used technique for improving image contrast. A successful fusion of the built environment and the natural landscape has already been improved using this technique for image contrast enhancement. The HE method equalizes the distribution of the values in a greyscale image. Every grey scale value should have nearly the number of equal pixels in the histogram, which should be distributed uniformly.

Histogram that has been flattened by replacing the original grayscale of a pixel *c* with the new grayscale *d* using the transformation function *R*. The equation $d=R$ can be used to express it mathematically *c*. An inverse translation can be extracted from as *s*, which is represented in Eq. (2).

2 $$c={R^{ - 1}}(d)$$

Here,$0<d<1$HE equalization is calculated with the help of Eq. (2)

3 $${M_0}=roun(\frac{{G{}_{{_{j}}}({2^r} - 1)}}{{Q,y}})$$

Here,${G_j}$ =distribution of cumulation from ${j^{th}}$ grayscale to the original image,

$roun$ =rotating the nearest value

${M_0}=$Value of gray level to histogram equalization

*Q* =image width

*y*= image height

Finally attained, a pre-processed image is indicated by$T_{i}^{{pre}}$.

## Rock hyraxes dandelion algorithm optimization-based optimal binary thresholding for breast tumor segmentation

### Rock hyraxes dandelion algorithm optimization

Comparison of Adam optimization technique: Tuning parameters is the key role of utilizing the optimization algorithm to get the optimal outcomes. Utilizing the Adam optimization technique^43^, it has the ability to adjust the learning parameters in the neural networks by effectively calculating the moving average of the gradients and squares. It shows faster convergence than the traditional gradient descent models by adjusting the parameters and provides robust performance. However, dealing with complex and smaller datasets leads to overfitting and also dealing with large amount of redundant data can leads to provide poor convergence.

The research work focus on RHSO, whereas it develops a multi-objective version and can tackle many optimization issues, including scheduling, parameter optimization, maintenance, and many others. These test functions are used to evaluate the exploitation and exploration capabilities of RHSO. Compared to other algorithms, it can better manage exploitation. A has good performance in unknown, difficult search spaces. Both algorithms provide benefits, including expanding the search space, a propensity for exploration, a tendency to approximate the global solution, and more. It quickly blossoms and produces large amounts of windborne seeds; this is one of the issues with dandelions. On the other hand, improved performance for models was created, and these algorithms are less efficient and have optimization issues. The proposed RHDAO is developed by combining two conventional algorithms: RHSO and DA.

The novelty of RHDAO algorithm: The hybrid algorithm known as RHDAO is suggested as a solution to this issue. Implementing the RHDAO algorithm is a powerful evolutionary model whereas, it ensures better convergence and prevents global optima issues. Selecting too large and too small a population count leads to high computational costs and limits providing better exploration capabilities. However, the developed RHDAO algorithm selects the essential number of population size to get valuable outcomes to ensure better robustness. As a result, the parameter *angle* is the new formulation in RHDAO is assessed by Eq. (4).

4 $$angle=\left( {\frac{{CF - BF}}{{WF - BF}}} \right) \times (360 - 0)+0$$

From the equation above, the terms for current fitness, best fitness, and worst fitness are *CF*, *BF* and *WF* respectively. *angle*Value is acquired and utilized to specify the circumstance. If the prerequisite is met, RHSO will upgrade the solution *angle* > 180; if not, DA will handle it. Thus, the following explains the mathematical models of two algorithms.

RHSO^44^: A small, fuzzy mammal called a rock hyrax (Procavia capensis) inhabits rocky terrain in sub-Saharan Africa and along the coast of the Arabian Peninsula. These mammals typically live in colonies where a single dominant male ferociously protects his area. Up to fifty people raise their children together, eat together, and even sleep together, and all play together. Three distinct species of hyrax exist; the first two are referred to as rock (or bush) hyrax, and the third is tree hyrax. It can be challenging to tell them apart in the field at a time. Rock hyraxes inhabit regions with a wide range of average temperatures as well as those that have enough food and water. The efficient extraction of the solitary stony protrusions through their dissemination may have been facilitated by fewer rates of metabolic and body of transparent temperatures.

Mathematical model and algorithm: Rock hyraxes begin spending many hours in the sun and sharing living spaces. They search for food collectively in a unique manner, forming a circle with various angles and dimensions. The Leader goes to a higher location to find food when they do.We shield one another from rapacious creatures. The population of the Rock hyrax swarm is initially made up of the leader and members. As was already established, the group’s leaders choose a high point from which to view the remainder of the group.

The Leader uses the calculation below to update his location based on his previous location.

5 $$Lead={l_1}*y(Lea{d_{posi}},j)$$

Where ${l_1}$ denotes a chance number between$[0,1]$, *y* denotes the Leader’s former position, $Lea{d_{posi}}$ denotes the Leader’s “old position, of *j* and *i* denotes “refers to each decrease” All members update their positions based on their position after the Leader’s position has been updated.

6 $$y(j,i)=(y(j,i) - (circle*y(j,i)$$

Where $circle$ denotes circular motion and attempts to emulate the circle system,

7 $$\begin{gathered} {m_1}={s_2}*\cos (angle) \hfill \\ {m_2}={s_2}*\sin (angle) \hfill \\ circle=sqr({m_1}^{2}+{m_2}^{2}) \hfill \\ \end{gathered}$$

Where $circle$ denotes circular motion and attempts to emulate the circle system, it is calculated as where ${s_2}$ is the radius and is a random integer between $[0,1]$and $angle$ denotes the angle of a move and is a random number between$[0,360]$. Every generation $angle$was also modified, and this modification was based on the lower and upper boundaries of the variable, where$BL$and $BU$denoted the lower and upper bounds of the random number generator.

8 $$delta=rand(BL,BU)$$

From Eq. (1), the $angle$ can be maintained within the predetermined range by setting it equal to 360 if the output value rises above 360 or equal to 0 if it falls below 0. Leader adjusting the position is provided in Eq. (1), followed by leader position, the other members adjusting the position, as illustrated in Eq. (1). Equation (2) evaluates each search agent’s fitness and chooses the best one to lead.

DA^45^: In DA, we assume that there are two sorts of the earth: those that are suited for dandelion sowing and those that are not, with dandelion living in both. Core dandelion (CD) refers to a suitable environment; helper dandelion refers to all other dandelion species (AD).

Take into account the following minimization problem without losing generality:

9 $$x=\hbox{min} h(y)$$

Finding an optimal y with the least amount of evaluation is the goal. Dandelion seeds are dispersed all around the dandelion when it is sowed. In our opinion, the dandelion seeding process can be considered as an attempt to find an optimal in a specific area surrounding a point. For instance, to discover a position y that satisfies$x=\hbox{min} h(y)$, we must first scatter the seeds of the dandelion in potential space otherwise find a point that is near to the point y. In DA, we must first choose n dandelions for each generation of planting; hence, we have m dandelions at this location. Computational Neuroscience and Intelligence to sow

Assuming that min and max are the minimum and minimum number of seeds, respectively, seeds ${N_j}$j for each dandelion ${M_j}$are determined as Eq. (10).

10 $${N_j}=\left\{ {\begin{array}{*{20}{c}} {\hbox{max} i \times \frac{{{h_{\hbox{max} i}} - h({y_j})+\varepsilon }}{{{h_{\hbox{max} i}} - {h_{\hbox{min} i}}+\varepsilon }}}&{{N_j}>\hbox{min} i} \\ {\hbox{min} i}&{{N_j} \leqslant \hbox{min} i} \end{array}} \right.$$

Where ${h_{\hbox{max} i}}=\hbox{max} i(h({y_i})$ and ${h_{\hbox{min} i}}=\hbox{min} i(h({y_i})$ also *ε* $\varepsilon$is the Epsilon of the machine it is used to reduce the value of the denominator and that is equal to 0.

According to Eq. (9), In comparison to a dandelion with a high fitness value, which will generate fewer seeds, the one with a low fitness value will produce more seeds. It cannot, however, be less than the necessary minimum of seeds. The two types of dandelions employed in DA are core dandelions and assistant dandelions; the core dandelion (CD), or the kind with the best fitness, is identified using Eq. (12).

11 $${y_{cb}}=\hbox{min} h({y_i})$$

The helper dandelions’ and the core dandelions’ radiuses are computed differently. Except for CD, the sowing radius of the assistant dandelions is determined by Eq. (13)

12 $$B_{i} (t) = \left\{ {\begin{array}{*{20}c} {BU - BL} & {l = 1} \\ {\omega \times B_{i} (l - 1) + (\left\| {Y_{{cd}} } \right\|_{\alpha } - \left\| {y_{i} } \right\|_{\alpha } )} & {otherwise} \\ \end{array} } \right.$$

From the above equation $BU$is the border of the upper function. And $BL$ is the border of the lower function. All dimensions are maximum which infinite norms are.

The helper dandelions’ seeding radius is set to the search space’s diameter at the start of the algorithm, starting from Eq. (9). This slowed the convergence rate and improved the performance of the global search. The design of the weight factor $\omega$ is as follows.

13 $$\varpi =1 - \frac{{he}}{{h{e_{\hbox{max} i}}}}$$

$he$is the evolution of the current function and $h{e_{\hbox{max} i}}$ is the function evaluation of maximum numbers. As can be observed, the value $\omega$changed from being enormous to being tiny. The issues of the radius sowing from the last generation on the recent sowing radius are decreasing.

However, it is a different method for calculating sowing radius for the CD, and it is created as follows.

14 $${B_{CD}}(l)=\left\{ {\begin{array}{*{20}{c}} {BU - BL} \\ {{B_{CD}}(l - 1) \times f\,\,} \\ {{B_{CD}}(l - 1) \times v} \end{array}} \right.\,\,\,\,\,\,\,\begin{array}{*{20}{c}} {l=1} \\ {b=1} \\ {b \ne 1} \end{array}$$

${R_{CD}}(l)$ is the CD’s generation sowing radius. *f* and *v* are the growth and withering factors, respectively, and a represents the growth trend, which is derived and which is shown in Eq. (15)

15 $$b=\frac{{{h_{CD}}(l)+\varepsilon }}{{{h_{CD}}(l - 1)+\varepsilon }}$$

**Fig. 3:**
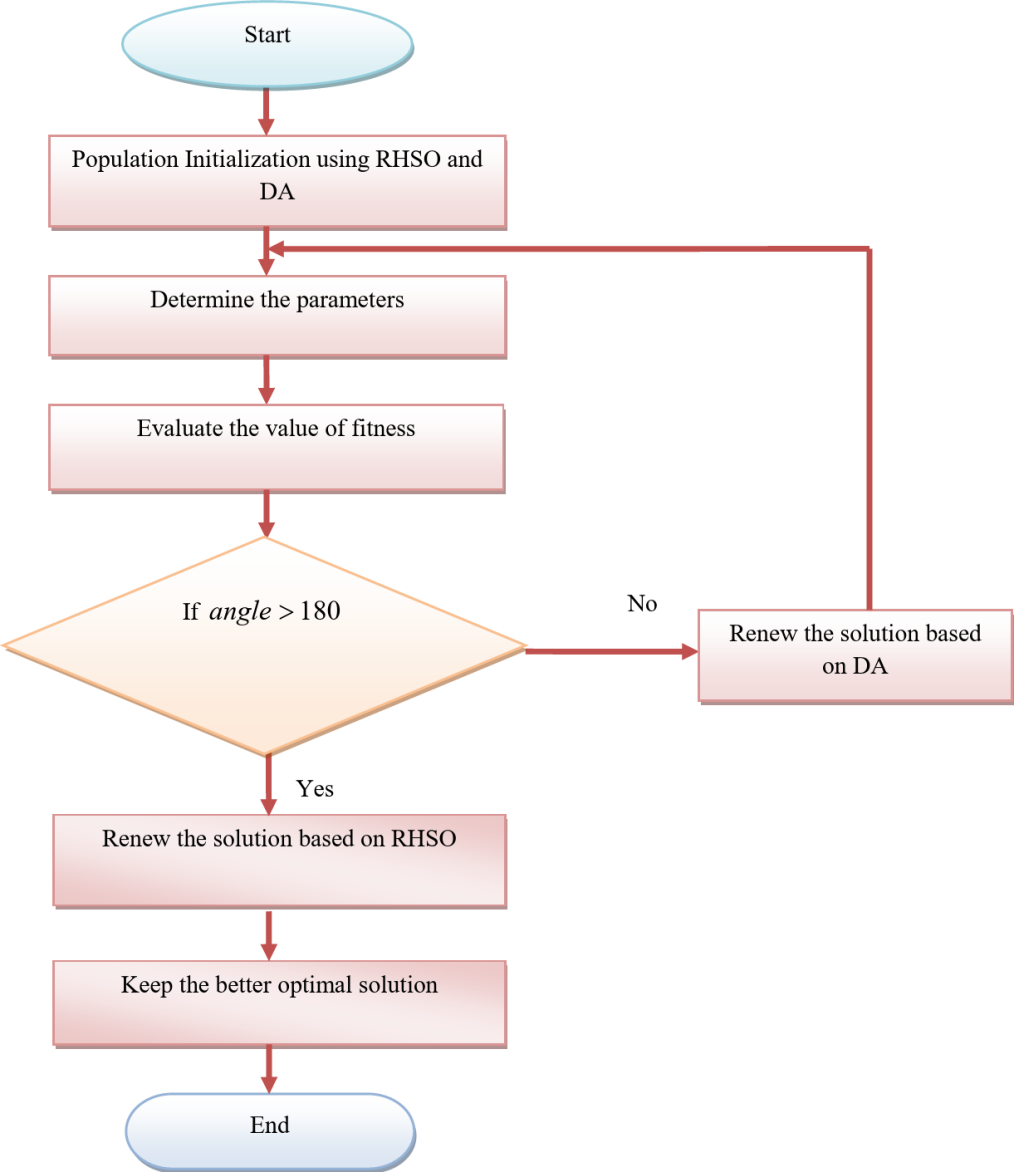
Flow diagram of proposed RHDAO.

Where the epsilon of the machine is used to avoid using a zero denominator. When $b=1$, it indicates that there is no better solution found by the algorithm and that the location is unsuitable. The factor withering *f* intended to depict the circumstance. *r* Cannot be so tiny; the values should be in the range of [0.8, 1). In contrast, when $b=1$, the algorithm finds a better solution than the previous generation, the location is ideal for the sowing radius and sowing, should be increased, based on which the growth factor e is proposed; obviously, *f* cannot be too great; the value is appropriate in [1, 1.1]. The search space’s lower and upper boundaries in dimension *k* are denoted by the terms${Y_{\hbox{min} {i^K}}}$and${Y_{\hbox{max} i{i^K}}}$. Fig 3 shows the flow diagram for the proposed RHDAO.

**Algorithm 1:**
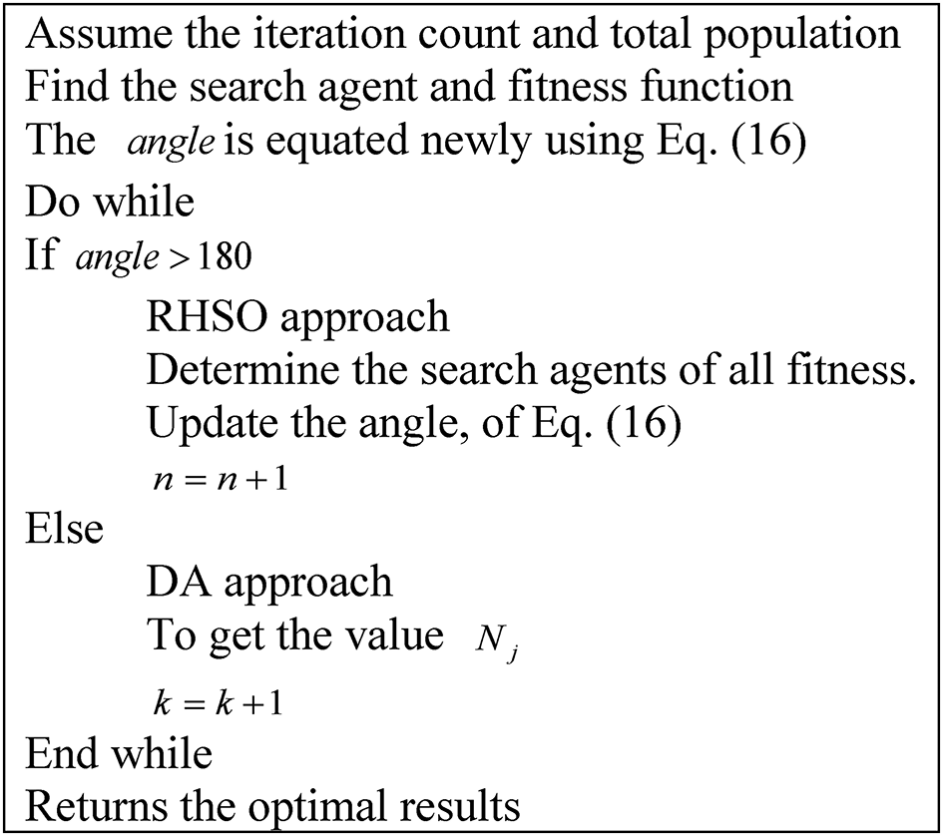
RHDAO.

The initial process begins by considering the number of population and iteration count by considering the behaviors in the RHSO and DA algorithm. Also, the fitness function is derived by utilizing Eq. (4). Here, the adoption of the fitness concept ensures to provide of potential solutions making the model more reliable and accurate. It enables to provide faster convergence in larger search space based on its specified criteria. Then, the condition $angle>180$ is derived to meet their requirements. If the given condition satisfies, the solution gets transferred into the RHSO algorithm in order to update the solution. Otherwise, it is given into the DA algorithm. This process is repeated based on the iterative process until it reaches the condition to get the desired outcomes.

### Optimal binary thresholding-based tumor segmentation

Thresholding is commonly used methods for segmenting images. It aids in separating the backdrop from the foreground. The fundamental aspect of thresholding performs with better computation efficiency. By choosing the proper threshold value, the grey-level image can be converted into a binary image. Commonly Otsu’s thresholding^46^ or adaptive thresholding^47^ has been widely applied in recent times. Unlike manual thresholding, Otsu’s thresholding is easy to implement in the segmentation process, in which it selects the automatic threshold selection. However, Otsu’s thresholding is sensitive to noise when dealing with different class sizes. Thus, it is unsuitable for complex images with varying of intensity levels. On the other hand, the adaptive thresholding is suitable for image segmentation. It is highly employed to perform with varying lighting conditions and different intensity distributions. However, the adaptive thresholding needs to be calculated with local thresholds that makes poor processing time. More replication takes place as the feature maps are combined with those of another layer. As a result, it generates the incorrect detection outcome. There is no guarantee that the thresholding method is recognizing adjacent pixels.

To overcome such issues, optimal binary thresholding is done using the RHDAO algorithm. Here, the population count of RHSO and DA algorithm is considered for initiating the process. The solution of threshold is encoded in the developed RHDAO algorithm. Here, the objective function for each threshold value is evaluated based on the fitness concept. Considering the RHDAO algorithm, it iteratively explores the larger search space by modifying the fitness concept and algorithmic rules. Convergence criteria are progressed with the help of iterative process until it reaches satisfactory outcomes. If the condition satisfies, the solution given into the RHSO algorithm otherwise, it given into the DA algorithm until the optimal solution is attained. Finally, the binary thresholding with highest fitness value is generally chosen as the optimal threshold by utilizing the developed RHDAO algorithm.

The preprocessing image is the input to binary thresholding$T_{i}^{{pre}}$.The resultant image of the binary thresholding shows whether the result is positive or negative. The resultant images vary from 0’s and 1’s.During pre-processing, the use of thresholding is to divide an image into smaller size, by defining their borders with at least one colour or greyscale value. Getting a binary image at the outset has the advantage of reducing the complexity of the data and simplifying the recognition and classification procedures. All pertinent information about the position and appearance of the items of interest must be included in the binary image *I*. The method used to convert a grey-level image into a binary image is the process of selecting a single threshold value *I*.

The most common method for turning a grey-level image into a binary image is the process of selecting a single threshold value *I*.

16 $$l(m,n) = \left\{ {\frac{{0h(m,n) < I}}{{1h(m,n) = > I}}\begin{array}{*{20}c} {} \\ {} \\ \end{array} } \right.$$

**Fig. 4:**
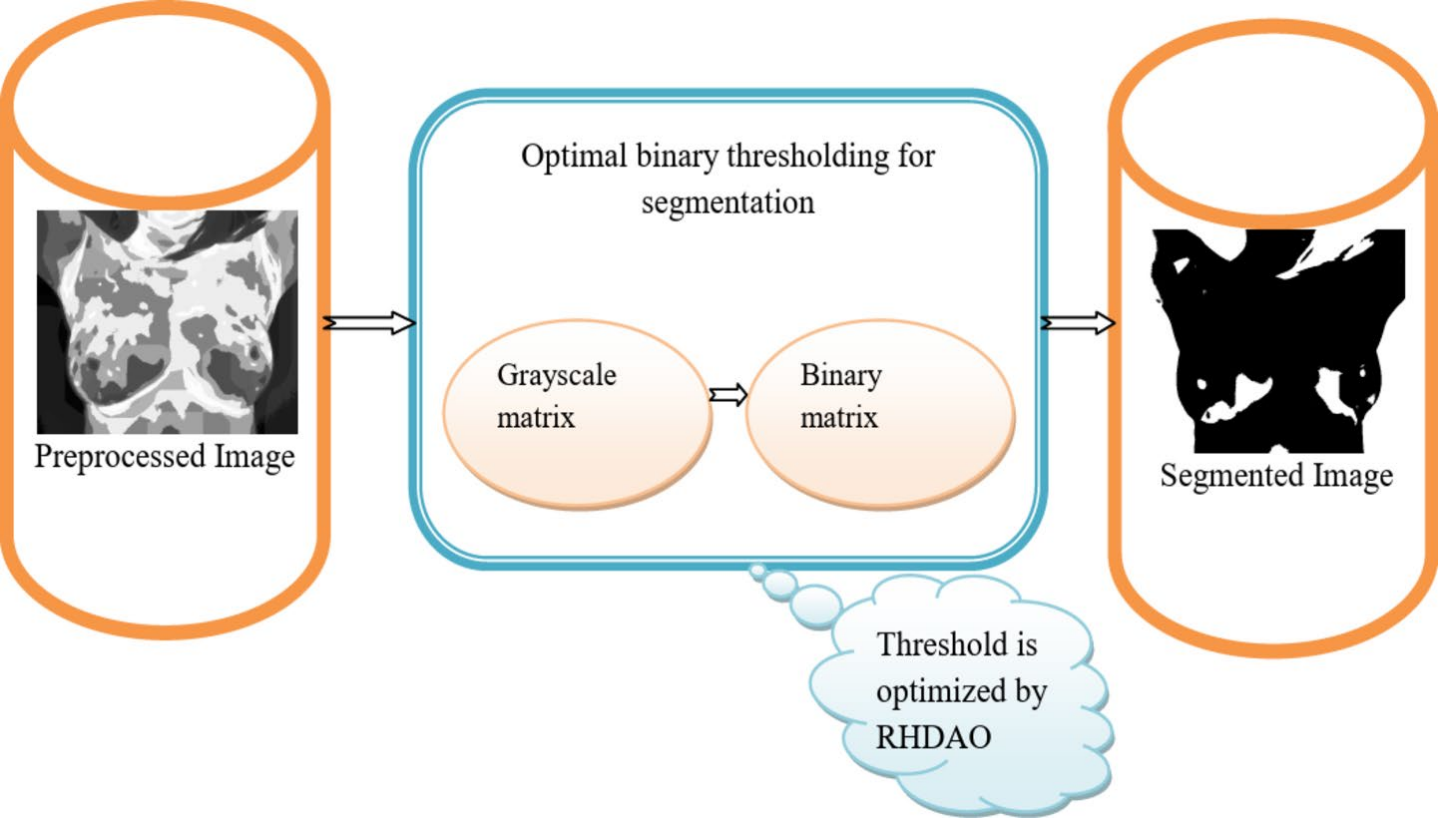
Architectural diagram for optimal binary thresholding.

Here$l(m,n)=o$, $l(m,n)$ the pixel of the foreground is 0 and $l(m,n)=1$ the pixel of the background is 1. Finally got the segmented image is denoted as${I_t}$.Here more complex application histogram. Fig 4 shows the schematic diagram for optimal binary thresholding in breast cancer classification.

### Proposed StackVRDNet with optimization

Preprocessing segmented image is given as input to the feature extraction technique. The extraction technique is defined as the algorithm ranking the features based on how well it can distinguish across classes. The number of features is then optimized to attain the highest classification accuracy rate using this optimization. In this proposed method segmented image is the input to VGG16, Resnet, and DenseNet.

**Fig. 5:**
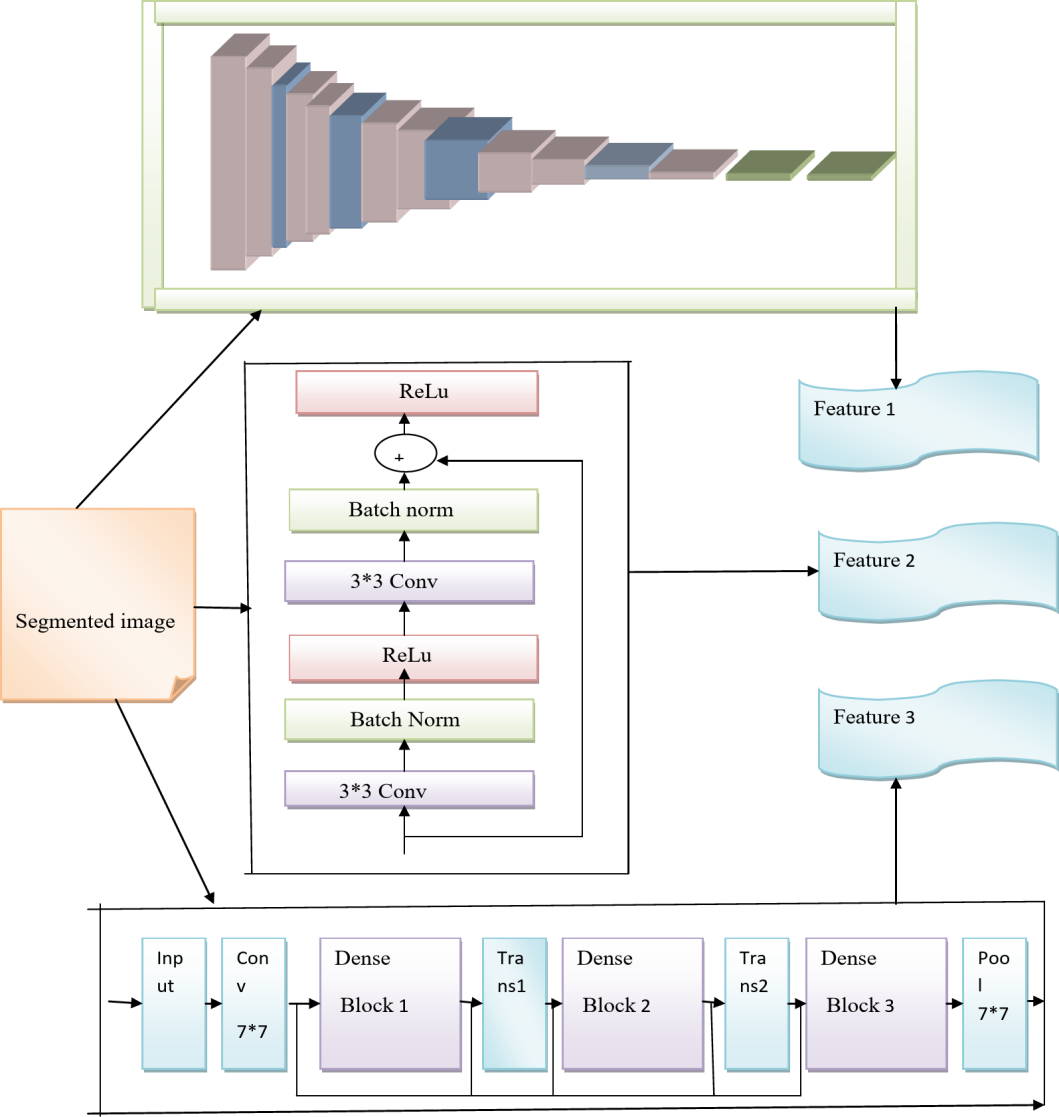
Diagram of proposed stackVRDNet with optimization.

Weight optimization of StackVRDNet in terms of convergence speed and stability: In the context of convergence speed, it enables to adjust the weight values in the stackVRDNet model that to learn a faster convergence rate during the process of training until it reaches the optimal solution by reducing the amount of training time in the network model. Assigning the appropriate weights in the stackVRDNet model ensures to maintain its stability in order to prevent from unwanted failures with specific conditions to reach its possible outcomes. While maximizing the stability, the assigning of unnecessary weights can maintain its structural integrity and prevents large fluctuations during the weight updates. The resultant features are used to fuse with the help of weight. It is accomplished by stacked weighted feature fusion. Here, the weight optimization is done by RHDAO. A stacked feature is the combination of three features with three weights finally it forms 3featuresets. Representing these featuresets as *f*_1_, *f*_2_ and *f*_3_ respectively. To find the stacked features combine the three features with three weights and form the weighted fused feature. These weights are tuned using RHDAO algorithm. Here, the epochs, activation layer and hidden neuron count are present in this optimization. DenseNet and VGG16 and ResNet are considered. In this method, epoch in VGG16, ResNet in the activation function, and DenseNet in the hidden count are tuned using the RHDAO algorithm. Improve this efficiency with StackVRDNet optimization. This increases the accuracy and easily identifies breast cancer. For the classification, these stacked features are given as input to VGG16, ResNet, and DenseNet and finally get output at three scores. In this classification, the final output provides highly ranked outcomes. Figure 5 shows the schematic diagram for the proposed stackVRDNet with optimization.

### Feature extraction phase

Reason for selecting feature extraction model: Extracting features is a crucial task for maximizing the detection performance. However, the conventional feature extraction model faces few pitfalls due to its heterogeneity in nature. Here, the breast tissue has varying densities and structures yet; the conventional feature extraction model fails to isolate the essential features within the images. Commonly, pre-trained models like Convolutional Neural Network (CNN), Recurrent Neural Network (RNN) and Deep Belief Network (DBN) are utilized for selecting the accurate features. For extracting the deep features in CNN, numerous data are required to get the significant features that limit the data availability. Understanding the CNN extraction process at each layer is still challenging, and it is difficult to provide better decision-making performance^48^. Capturing the long term dependency in the sequential data provides challenges that often increase the issue of vanishing gradient thus; it limits the feature extraction performance using RNN^49^. Due to its complex architectural structure, the RNN model potentially extracts irrelevant features that significantly impact the model’s performance. Focusingon the DBN model, it often relies on noisy or biased data affects generalization performance and shows irrelevant features^50^.

In order to get accurate features, the research work employs VGG16, ResNet and DenseNet models for maximizing the performance. This section explores the feature extraction process, where the input is taken as a segmented image. The three various techniques are utilized to extract the features that are illustrated below.

VGG16^51^: The input of this VGG16 is a segmented image. The VGG16 network was built using incredibly tiny convolutional filters. It was built using three fully linked layers and 13 convolutional layers. It employs a simply constructing of the system. The VGG16 network primarily consists of a deep convolution neural network that was created with consideration for the proper layer depth settings that don’t enhance the network’s complexity. Additionally, the VGG network was able to decrease training loss and save more data for object detection as a result of the addition of additional layers. However, the VGG16 model is expensive for evaluating lots of resource constraints. A million parameters make up VGG16. The SVM classifier replaces the fully connected layers, where the majority of these parameters are located. The output of this VGG16 results in the featuresets *f*_1_.

ResNet^52^: The input that has been given to ResNet is a segmented image ResNet is accurate to a high degree. With 152 layers and a distinctive architecture that introduces residual blocks, the network is extremely deep. It deals with the problem of employing identity skip connections to train a deep architecture. The inputs from the layers are copied by these leftover blocks and forwarded to the following layer. The disappearing problem is resolved by the identity to skip the connection step. This architecture was created to overcome problems with deep learning training since deep learning training is time-consuming and has a restricted number of layers. The design of the ResNet model is simple and easy to implement. By excluding connections on two to three levels that contain Rectified Linear Units (ReLU) and batch normalization between the architectures, the ResNet model is implemented. Stochastic Gradient Descent (SGD) is used to initialize the ResNet weights of conventional momentum parameters. The Expression of this ResNet is shown in Eq. (16).

17 $$y=F(n,{W_2}+n)$$

From this above equation the output of the Resnet is considered as a featureset${f_2}$, ${W_2}$is the weight of the ResNet and *F* is the function of this feature.

**Fig. 6:**
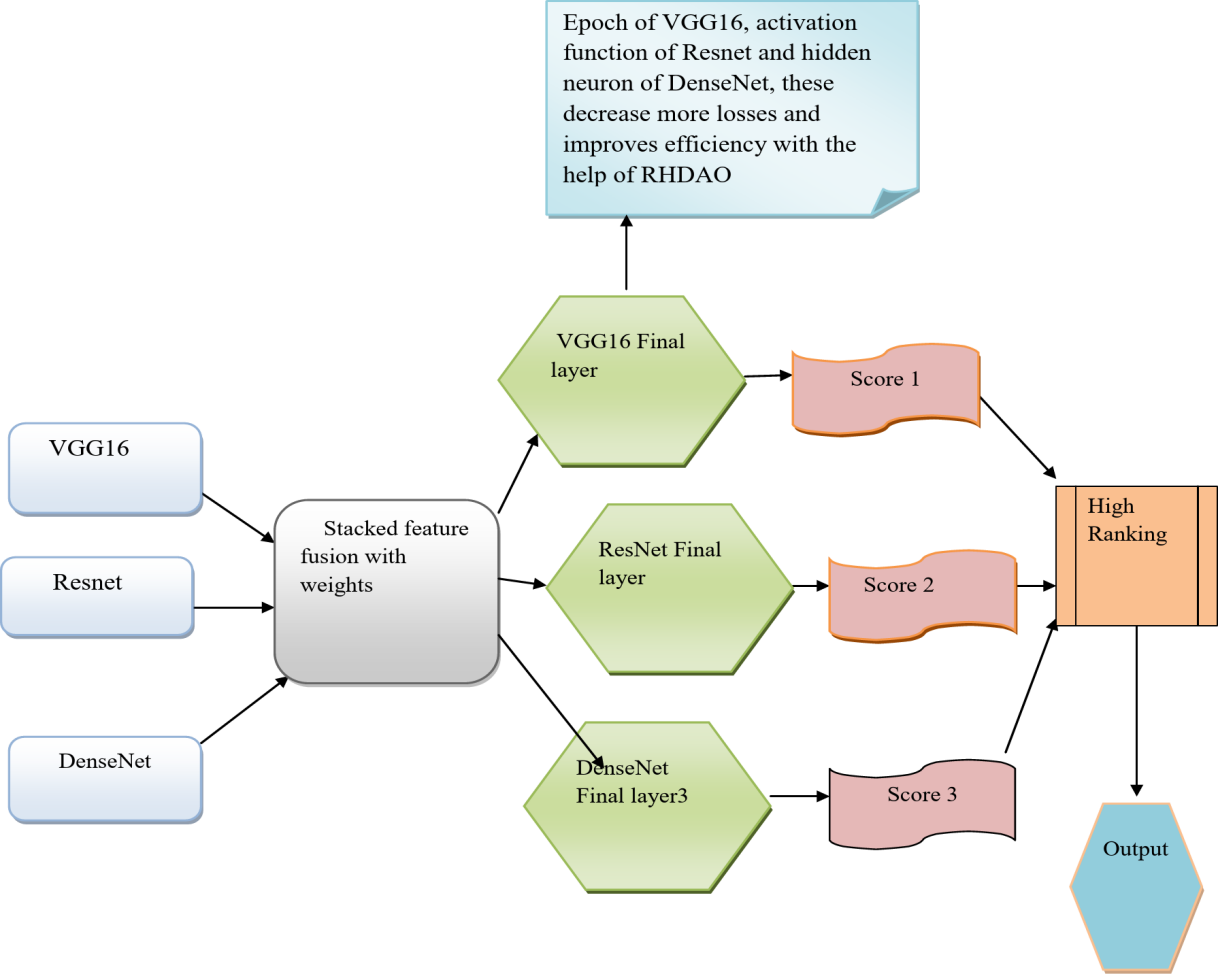
Diagram for the feature extraction phase.

DenseNet^53^: The input that has been given to DenseNet is a segmented image. The images are subjected to a detection technique after they have been gathered. The DenseNet model is utilized to detect abnormalities. DenseNet is the new architecture used by the convolutional-based deep learning method. The model is based on connecting the convolutional layers that are displayed in each dense block. It considerably helps in the detection of anomalous occurrences in photos because it is feature-based. To estimate the features, which are concatenated and given to each layer as input, each layer’s parameters are also trained. In this instance, the input is the gathered image. Below is a description of it, which is mostly made up of “a dense block and transition layer. A fully connected layer, a softmax classifier, and alternately dense and transition blocks are used to build the simplified DenseNet. This dense block consists of a batch normalization (BN) layer, a convolutional layer, and a Leaky ReLU (LReLU) layer the layers that make up the two cascaded convolutional units. The transition block’s purpose is to determine an efficient composition among feature maps created from various between the final transition block and the FC layer. The Global Average Pooling (GAP) approach is used to prevent flattening the final output convolutional layer and prevent the introduction of a significant number of weights. Additionally, the proposed hybrid network is more robust because the GAP technique can be utilized to examine the network’s final feature maps. The output of this denseness results in the featuresets${f_3}$. Fig 6 shows the schematic diagram for the feature extraction phase.

Combining process of VGG16, ResNet, and densenet with stacked weighted feature pool: In this proposed method, the segmented image is inputted into VGG16, ResNet, and DenseNet models. VGG16 model helps to effectively extract the hierarchical features from the segmented image based on its deep structure. By adopting multiple convolutional layers in VGG16, the fine details from the complex image get extracted to enhance robustness performance. The ${f_1}$featureset is attained in the VGG16 model. Considering skip connection in ResNet model facilitates to extract fine details and mitigates from vanishing gradient problem. Capturing the intrinsic features from the ResNet model facilitates to improve the accuracy of the model and provides better diagnosis in clinical settings. The second feature set of ResNet model is attained as${f_2}$. With the help of dense connections in the DenseNet model, the number of parameters gets significantly reduced to emphasize a better flow of feature representation especially in complex datasets. Each layer in DenseNet model can allow learning the features earlier for extracting the richer features. The final featureset of DenseNet model is taken as${f_3}$. The output from each model is given into the stacked weighted feature pool. Stacked weighted Feature is a crucial data preprocessing technique that can get rid of certain redundant or unneeded features and increase the effectiveness and precision of classifiers. It is also improving the stacking-based categorization model and information-gathering systems. After processing the output of these three models, it provides a fused feature. Representing these featuresets as${f_1}$,${f_2}$ and ${f_3}$respectively. The weights${W_1}$,${W_2}$and${W_3}$, were assigned to each featureset. To find the stacked features combine the three features with three weights. This stacked feature is derived as and shown in Eq. (17).

18 $$SF=({W_1}*{f_1})+({W_2}*{f_3})+({W_3}*{f_3})$$

Finally, these acquired features are given to VGG16, ResNet, and DenseNet. Term$SF$as the stacked feature and *w* is the weight and *f* is considered a feature.

### Classification phase

In this classification phase, the features are extracted from three models they are VGG16, Resnet, and DenseNet. The three features are explained below.

VGG16: The input for VGG16 is the stacked feature. The output of this feature is provided by the last layer. One of the main drawbacks of VGG16 network is huge and consumes more time for training the model. The VGG16 model is larger than 533 MB due to the model’s depth and the number of entirely connected layers. The parameter used in this VGG16 is Epoch. It boosts only certain limits. To overcome these issues these features are optimized using RHDAO.

ResNet: The second feature is ResNet. The input given to ResNet is a stacked feature. The drawback of this ResNetis a deeper network often takes weeks to train, which makes it nearly impossible to use in real-world applications. A parameter used in this feature is the activation function. To overcome these issues which are optimized using RHDAO?

DenseNet: At last stacked feature is the input to DenseNet. The drawbacks of this DenseNet are each layer’s feature maps are combined with the one before them, and the data is repeated several times. The parameter used in this DenseNet is hidden count. To improve this accuracy and efficiency this is optimized using RHDAO.

The proposed classification objective function of the model is derived using Eq. (31).

19 $$Obj=\mathop {\arg \hbox{max} }\limits_{{\left\{ {{W_{1,}}W,{W_3},C{N^{EP}},D{V^{ED}},E{L^{DN}},TH} \right\}}} \left[ {ay} \right]$$

The terms like hidden neuron count, activation layer and epochs. Epoch is denoted as$C{N^{EP}}$the Hidden layer is represented $D{V^{ED}}^{{}}$ and the activation layer $E{l^{DN}}$is taken for optimization. The range of the hidden neuron count is [5, 255], the epoch count is limited to between 50 and 100, and there are three weights in the stack VRDN network for the activation function. The thresholding varies from 200 to 255. The term$ah$ defines the accuracy value that is denoted by a “closed value concerning true measure”. It is shown in by Eq. (19)

20 $$ah=\frac{{nrA+nrB}}{{nrA+nrB+laA+laB}}$$

**Fig. 7:**
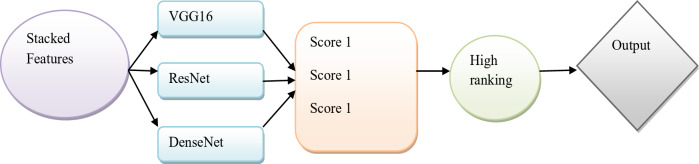
Diagram for classification phase.

In the above expression, the variable $$nrA$$$$nrB$$specifies the “True Positive (TP) and True Negative (TN)” rates as well as the term $$laA$$and $$laB$$signifies the “False Positive (FP) and False Negative (FN)”. Fig 7 shows the proposed classification phase for breast cancer.

## Results and discussion

### Experimental settings

The Python platform is performed to run the suggested breast cancer classification model, and several metrics were utilized to estimate the performance using PyCharm community Edition 2023.3.7. Thus, the existing algorithms like Human Felicity Algorithm (HFA)-StackVRDNet^54^ and Small World Optimization Algorithm (SWOA)-StackVDRNet^55^, RHS-StackVRDNet^44^ and DA-StackVRDNet^45^ were taken. Moreover, the conventional models were considered as CNN + EfficientNetV2B3^21^, DCNN^24^, DLF^18^, and StackVRDNet^54^, respectively, and it also carried out the results. 25 maximum iteration counts are in the proposed algorithm.

21 $${A_s}=\frac{{nrA}}{{nrA+{l_{}}aB}}$$

Specificity: “Specificity is measured by the probability of negative rate”.

22 $$spec=\frac{{nrB}}{{n{r_{}}B+laB}}$$

FPR and FNR: It estimates the observation that is wrongly detected. On the other hand, the False Negative Rate calculates the anomalies inaccurately even if it contains the images.

23 $$FCR=\frac{{laA}}{{laP+naA}}$$

24 $$FSP=\frac{{nrB}}{{laN+nrA}}$$

Recall: Recall is used to evaluate the number of actual positive values.

25 $$Rm=\frac{{nrA}}{{nrA+laB}}$$

F1-Score: The harmonic values of recall and precision is known as the FI-score.

26 $$FIScore=2*{\frac{{Ak*Rn}}{{Ak+Rn}}_{}}$$

FDR: False Discovery Rate is determined by estimating the ratio of FP and both TP and FP.

27 $$FDR=\frac{{laA}}{{nrA+laA}}$$

NPV: Negative Predictive Rate is evaluated by the ratio between TN and both TN and FN.

28 $$NPV=\frac{{NrB}}{{nrB+laB}}$$

MCC: It denotes the difference among the detected image output and actual image.

29 $$MCC=\frac{{nrA \times nrB - laA \times laB}}{{\sqrt {\left( {nrA+laA} \right)\left( {nrA+laB} \right)\left( {nrB+laA} \right)\left( {nrB+la} \right)} }}$$

#### Addressing class imbalance issues during training/testing

Some of the models are not effectively learn about the minority class leads to poor performance and data imbalance issues. Increasing data imbalance issues affect the accuracy sometimes, it shows misclassification outcomes. The imbalanced dataset can resultto be poor generalization ability on the unseen data making the model inaccurate. In order to solve these issues, at first the selection of appropriate metrics enables handling the data imbalance issue during training/testing. For instance, focusing accuracy as the total number of accurate predictions by the total number of predictions. By understanding the complexities of data imbalance issues, implementing the deep learning model can effectively manage these issues to provide accurate performance and shows better decision-making performance in real-world scenarios.

### Imaging results for both processed image and preprocessed image

**Fig. 8:**
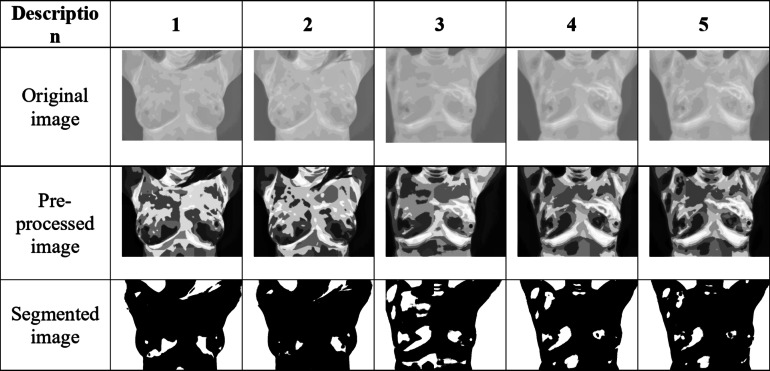
Preprocessed and segmented resultant image.

Figure 8 shows the resultant images by applying the novel detection of breast cancer.

#### Model interpretability using Grad-CAM techniques

**Fig. 9:**
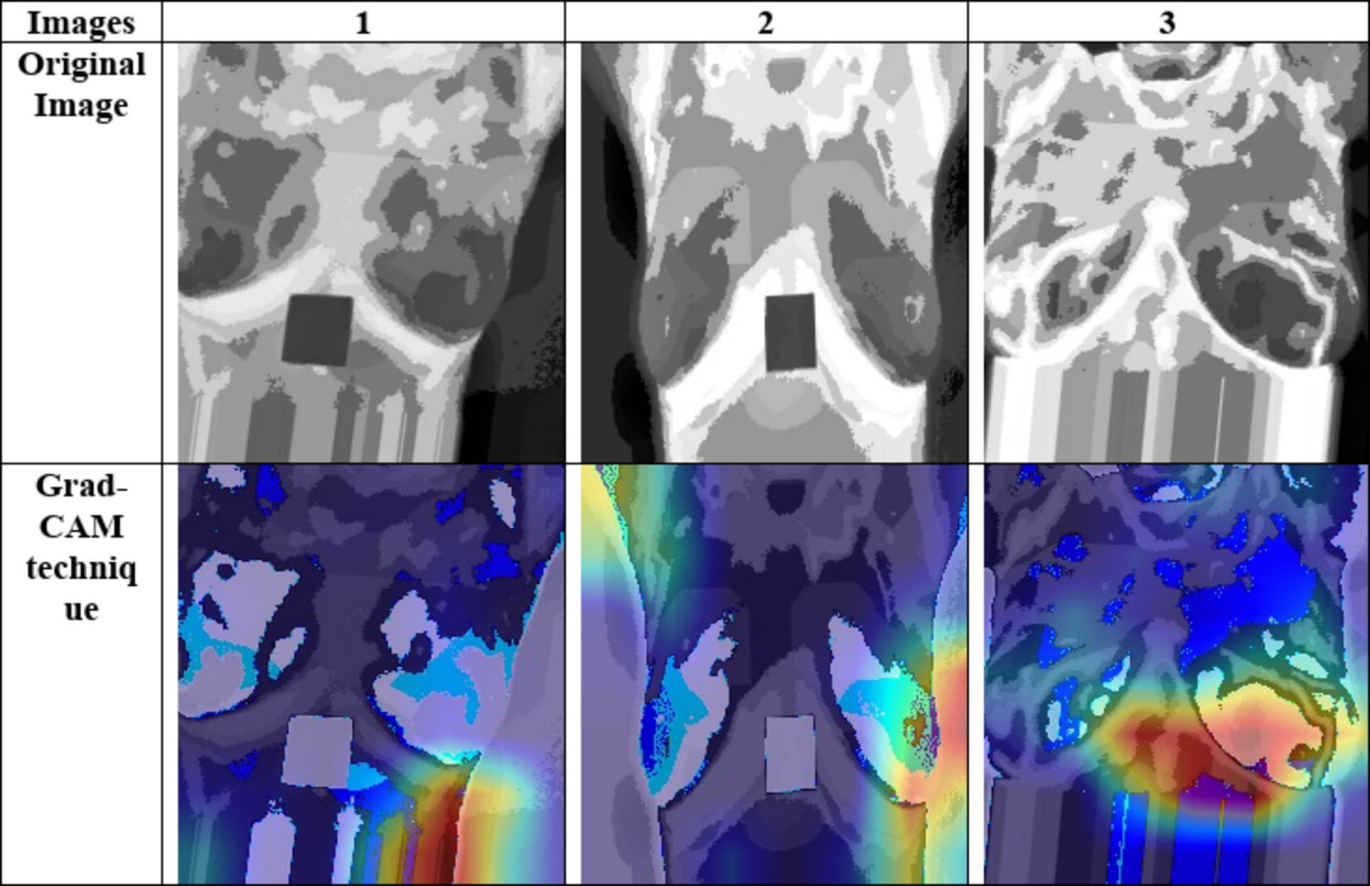
Analysis of model interpretability using Grad-CAM techniques.

In order to provide a better visualization outcome, the Grad-CAM technique is compared in Fig. 9.Gradient-weighted Class Activation Mapping (Grad-CAM)^56^ model helps to provide better visualization outcome to enhance the prediction performance and relevant diagnosis. It produces a clear visual representation using the gradients in the final model layer highlighting the important image regions. Formally, the Grad-CAM model is generalized for many CNN architectures. Moreover, the Grad-CAM model is applicable in visual questioning answering system, image classification and image captioning. Here, the key proficiency of utilizing the Grad-CAM model boost up the resolution of the image in complex medical images. Enhancing the image quality suggest to provide accurate decision-making performance in clinical settings. Thus, it ensures to improve the interpretability and trust performance that tends to maximize clinical decision-making performance.

### Performance analysis of existing methodologies in breast cancer detection

**Fig. 10:**
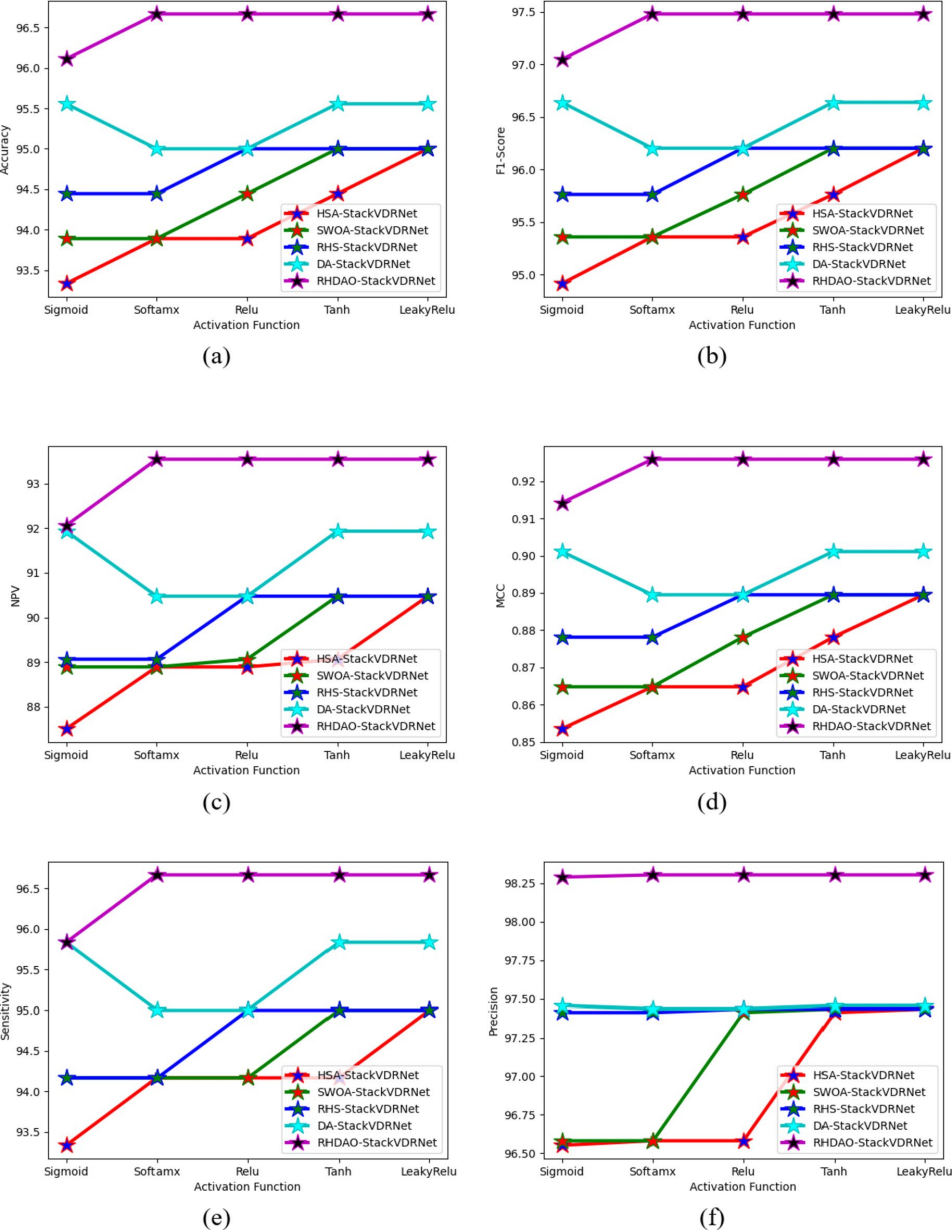

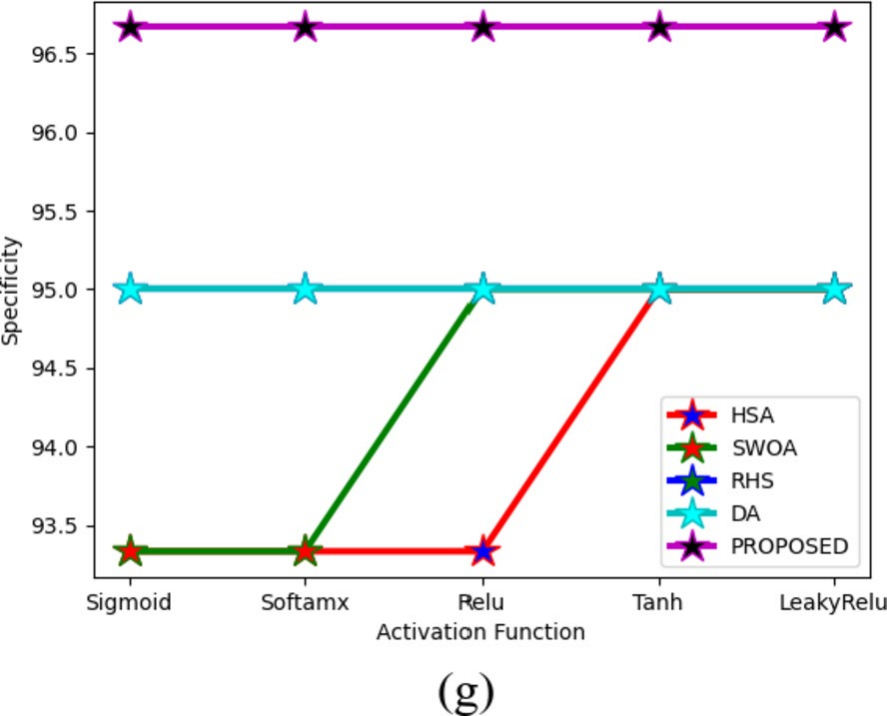
Activation function-based analysis of proposed breast cancer diagnosis model compared with traditional algorithms in terms of (**a**) Accuracy, (**b**) F1-score, (**c**) NPV (**d**) MCC, (**e**) sensitivity, (**f**) precision and (**g**) specificity.

**Fig. 11:**
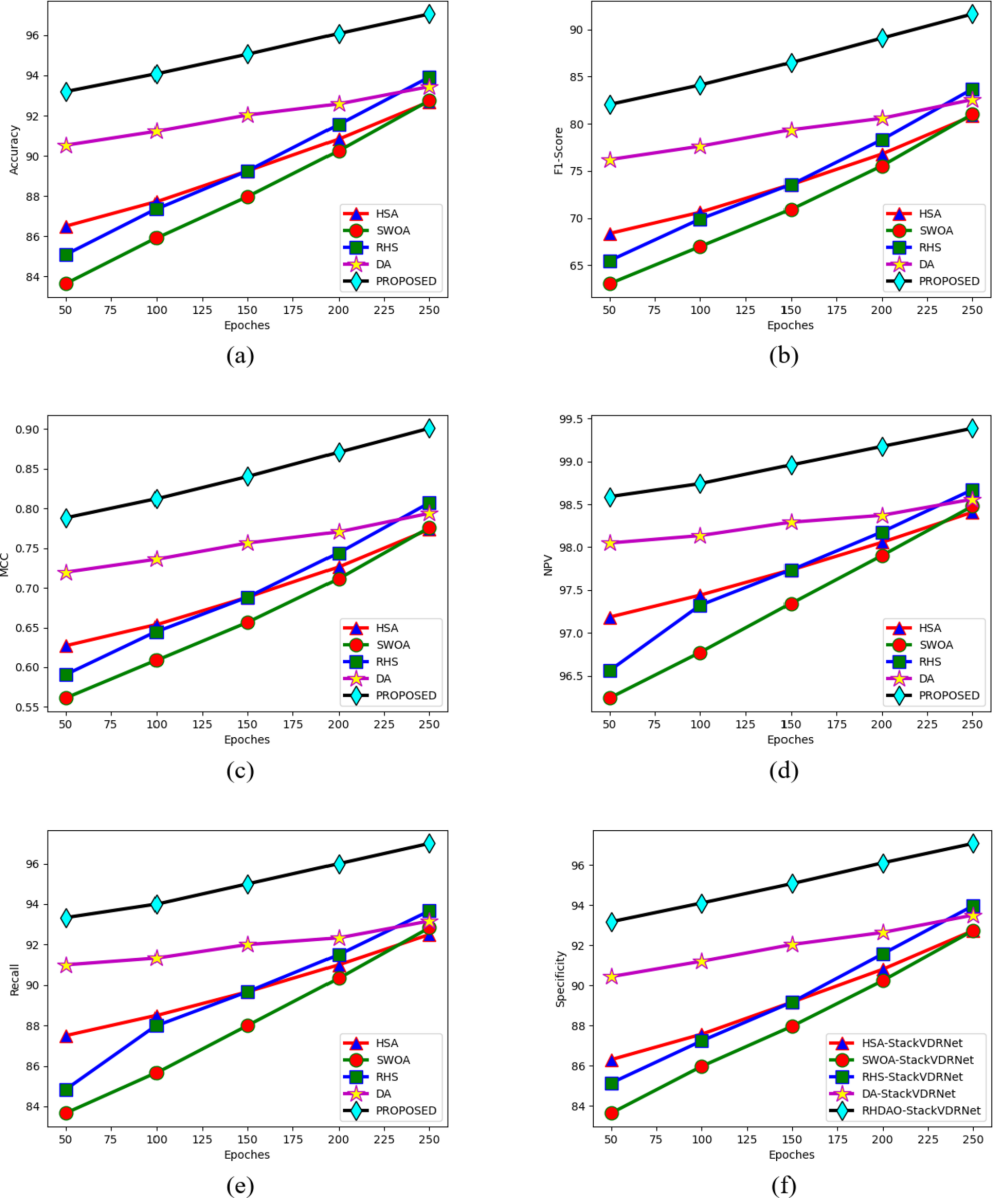
Epoches-based analysis of proposed breast cancer diagnosis model compared with traditional algorithms in terms of (**a**) Accuracy, (**b**) F1-score, (**c**) MCC, (**d**) NPV, (**e**) Recall, and (**f**) specificity.

The effectiveness of the suggestive approach in comparison to other algorithms is presented in Figs. 10 and 11 for two datasets. The proposed method’s NPV analysis is shown in Fig. 11c. The HSA-StackVDRNet, SWOA-StackVDRNet, RHS-StackVDRNet, DA-StackVDRNet, and RHDAO-StackVDRNet obtained FI-score values of 41, 16, 41, and 46% respectively, which is better than the RHDAO-StackVDRNet proposed in accordance. Validating precision measures, the diverse activation function is compared and validated by existing baseline models. In addition to these, the existing HsA-StackVDRNet model shows lower performance in all considered activation functions. This poor performance can limit from providing better diagnosis treatment. Poor precision analysis can leads to a high rate of false positives shows the absence of cancer. Thus, it creates complexities during the treatment. Validating F1-score analysis, the existing DA-StackVDRNet model shows a second better performance compared to the other existing approaches. However, the developed RHDAO-StackVDRNet model shows better performance by neglecting the false positives and false negatives to minimize the false outcomes. This makes the developed model more reliable and provides accurate performance in the breast cancer diagnosis process. Therefore, the greater the F1-score shows better diagnosisand detect anomalies in the image.

### Performance analysis of existing methodologies in breast cancer detection

**Fig. 12:**
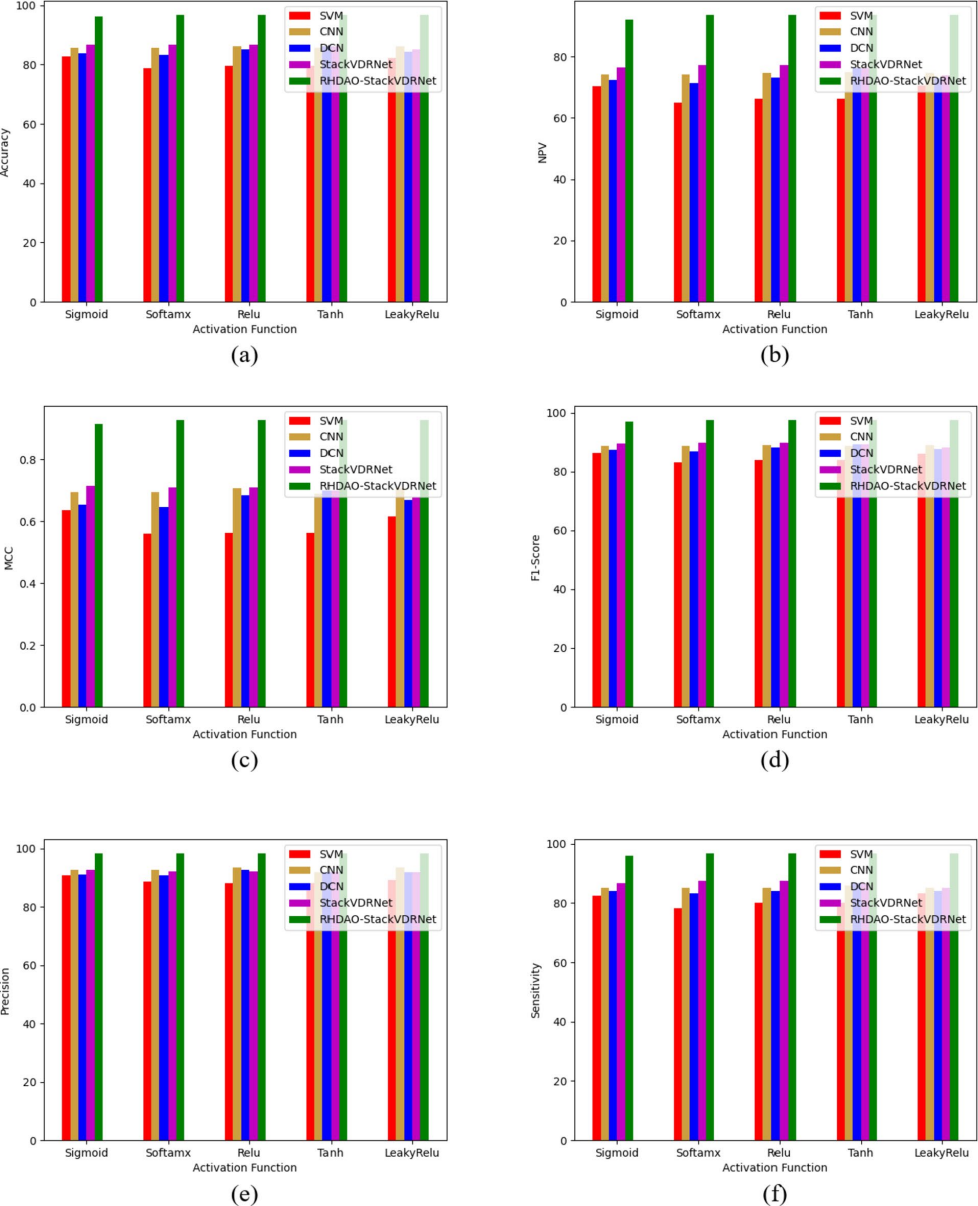

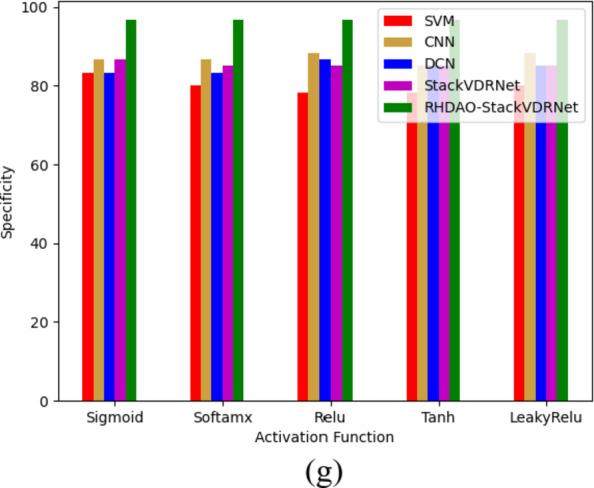
Performance-based analysis of proposed breast cancer diagnosis model compared with traditional algorithms in terms of (**a**) Accuracy, (**b**) NPV, (**c**) MCC, (**d**) F1-score, (**e**) precision, (**f**) sensitivity and (**g**) specificity.

**Fig. 13:**
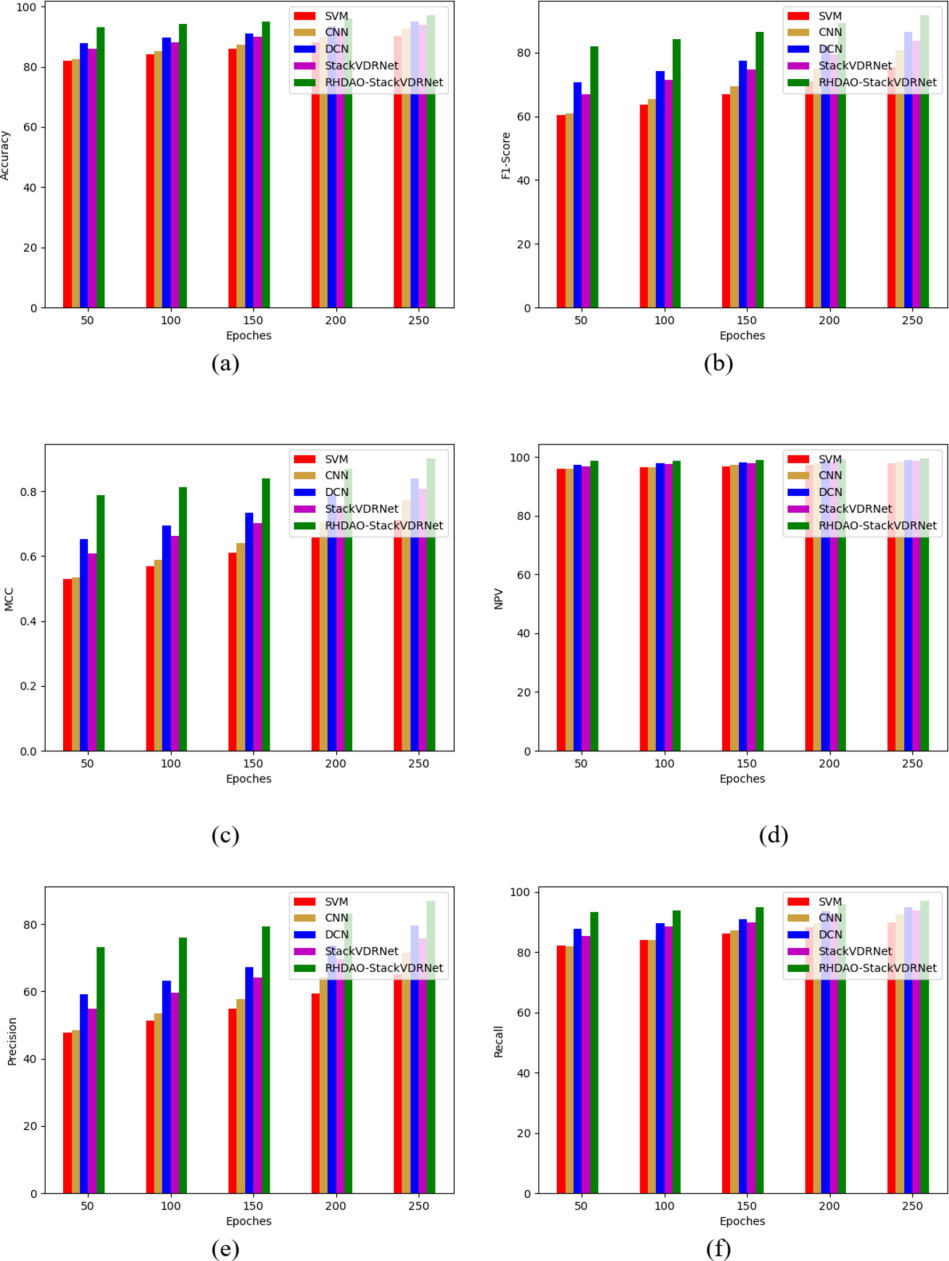

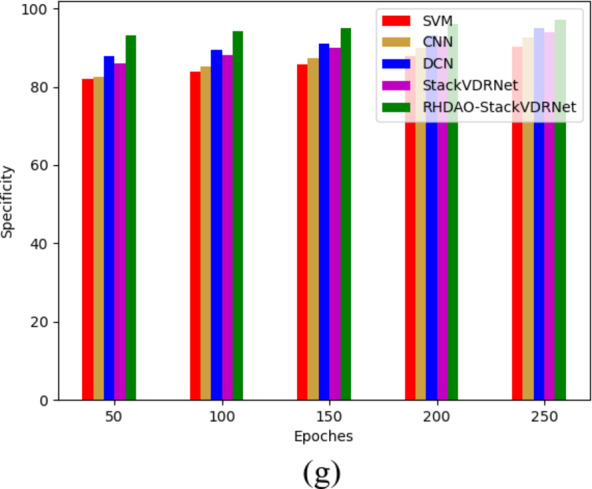
Performance-based analysis of proposed breast cancer diagnosis model compared with traditional algorithms in terms of “(**a**) Accuracy, (**b**) F1-score, (**c**) MCC (**d**) NPV (**e**) precision (**f**) RECALL and (**g**) specificity.

The performance study of the novel model in comparison to traditional optimizations and learning models is shown in Figs. 12 and 13 for two datasets. Diverse performance measures are taken for the validation, in which it provides accurate performance in the designed method. Considering precision and F1-score measures, it is the crucial measure to identify the cancerous cases over the non-cancerous cases. Analyzing the existing models is ineffective while dealing with imbalanced datasets and leads to provide unnecessary decisions, which impact the overall system performance. On the other hand, the performance of the developed RHDAO-StackVDRNet model shows accurate performance whereas the medical professionals can leads to provide better diagnosis treatment and prevent from cancer at earlier stage. Accordingly, the proposed work achieves a precision rate that is 37% greater than SVM, 41% higher than CNN, 39% higher than DCN, and 41% more advanced than StackVDRNet. As a result, the more MCC findings there are, the more effectively they can detect aberrant events in photos.

### The ROC analysis of existing methodologies in breast cancer detection

**Fig. 14:**
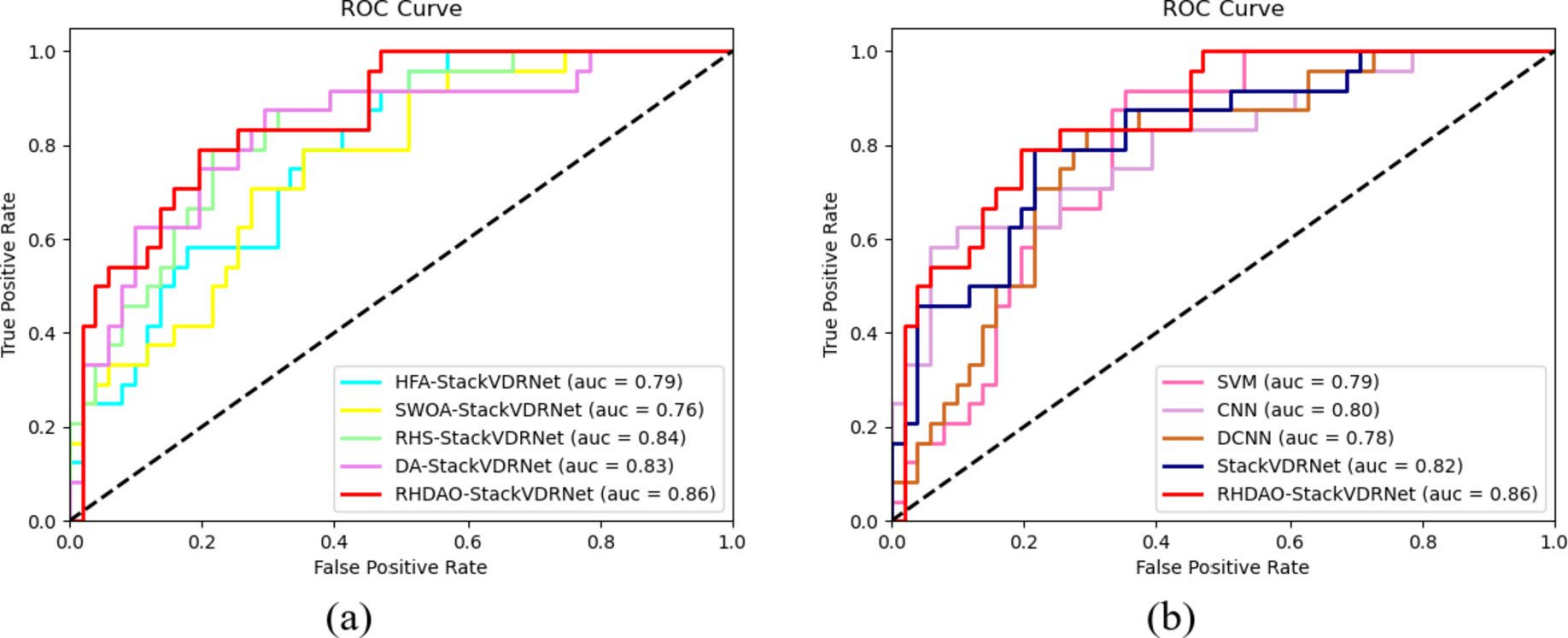
ROC analysis of proposed breast cancer diagnosis model compared with traditional algorithms in terms of “(**a**) Algorithm comparison, (**b**) Classifier comparison.

For dataset 1, the suggested method’s ROC performance was compared with that of several methods (provided in Fig. 14a) and conventional classifiers (shown in Fig. 14b). The True positive rate of the proposed technique is shown in Fig. 14a. The True positive rate values obtained by HFA-StackVDRNet, SWOA-StackVDRNet, RHS-StackVDRNet, and DA-StackVDRNet are 34, 29.2, 18.8, and 13.6%, respectively. Because of this, the True positive rate guarantees the system’s effectiveness in spotting anomalies in photos.

### The overall analysis of the suggested method in contrast with traditional algorithms

The overall evaluation of the model compared with various algorithms and classifiers is given in Tables 2 and 3. The recall of the proposed RHDAO-StackVRDNet model obtains a higher value than 4.7, 4.6, 3.3, and 3.8% by HFA-StackVDRNet, SWOA-StackVDRNet, RHS-StackVDRNet, and DA-StackVDRNet. These findings have declared the system is proficient to detect breast cancer.

**Table 2:** Performance analysis over existing recommended breast cancer classification model compared with traditional learning models.

Metrics	HFA-StackVDRNet^41^	SWOA-StackVDRNet^42^	RHS-StackVDRNet^44^	DA-StackVDRNet^45^	RHDAO-StackVDRNet
Accuracy	92.694	92.750	93.917	93.444	97.056
Recall	92.500	92.833	93.667	93.167	97.000
Specificity	92.733	92.733	93.967	93.500	97.067
Precision	71.798	71.871	75.639	74.138	86.866
FPR	7.267	7.267	6.033	6.500	2.933
FNR	7.500	7.167	6.333	6.833	3.000
NPV	98.408	98.478	98.670	98.559	99.386
FDR	28.202	28.129	24.361	25.862	13.134
F1-Score	80.845	81.018	83.693	82.570	91.654
MCC	0.774	0.776	0.807	0.794	0.901

**Table 3:** Performance analysis over existing recommended breast cancer classification model compared with traditional learning models.

Metrics	CNN+EfficientNetV2B3^21^	DCNN^24^	DLF^18^	StackVDRNet	RHDAO-StackVDRNet
Accuracy	90.27778	92.63889	95.08333	93.94444	97.05556
Recall	90	92.5	95	93.83333	97
Specificity	90.33333	92.66667	95.1	93.96667	97.06667
Precision	65.06024	71.6129	79.49791	75.67204	86.86567
FPR	9.666667	7.333333	4.9	6.033333	2.933333
FNR	10	7.5	5	6.166667	3
NPV	97.83394	98.40708	98.95942	98.70448	99.38567
FDR	34.93976	28.3871	20.50209	24.32796	13.13433
F1-Score	75.52448	80.72727	86.56036	83.77976	91.65354
MCC	0.710809	0.772228	0.840774	0.8081	0.900743

### Statistical results of the proposed method compared with different optimizations

Table 4 elucidates the statistical analysis of the proposed model in contrast with classical optimizations for all three datasets. These calculations are done by five distinct metrics. “The best and worst values are the maximum and minimum rates. The median value is the mid value, whereas the mean refers to the average value. Finally, the standard deviation is defined as the degree of deviation between each execution.”. As a result, the table findings make sure the system gets more encouraging readings to find abnormalities and locate them in images.

**Table 4:** Statistical analysis of existing recommended breast cancer classification model compared with traditional learning models.

Metrics	HFA-StackVDRNet^41^	SWOA-StackVDRNet^42^	RHS-StackVDRNet^44^	DA-StackVDRNet^45^	RHDAO-StackVDRNet
Best	1.002	1.004	1.001	1.002	1.000
Worst	1.003	1.036	1.168	1.161	1.069
Mean	1.002	1.011	1.010	1.024	1.008
Median	1.002	1.004	1.002	1.023	1.000
Std	0.001	0.009	0.033	0.030	0.015

### Performance analysis of thresholding techniques

**Fig. 15:**
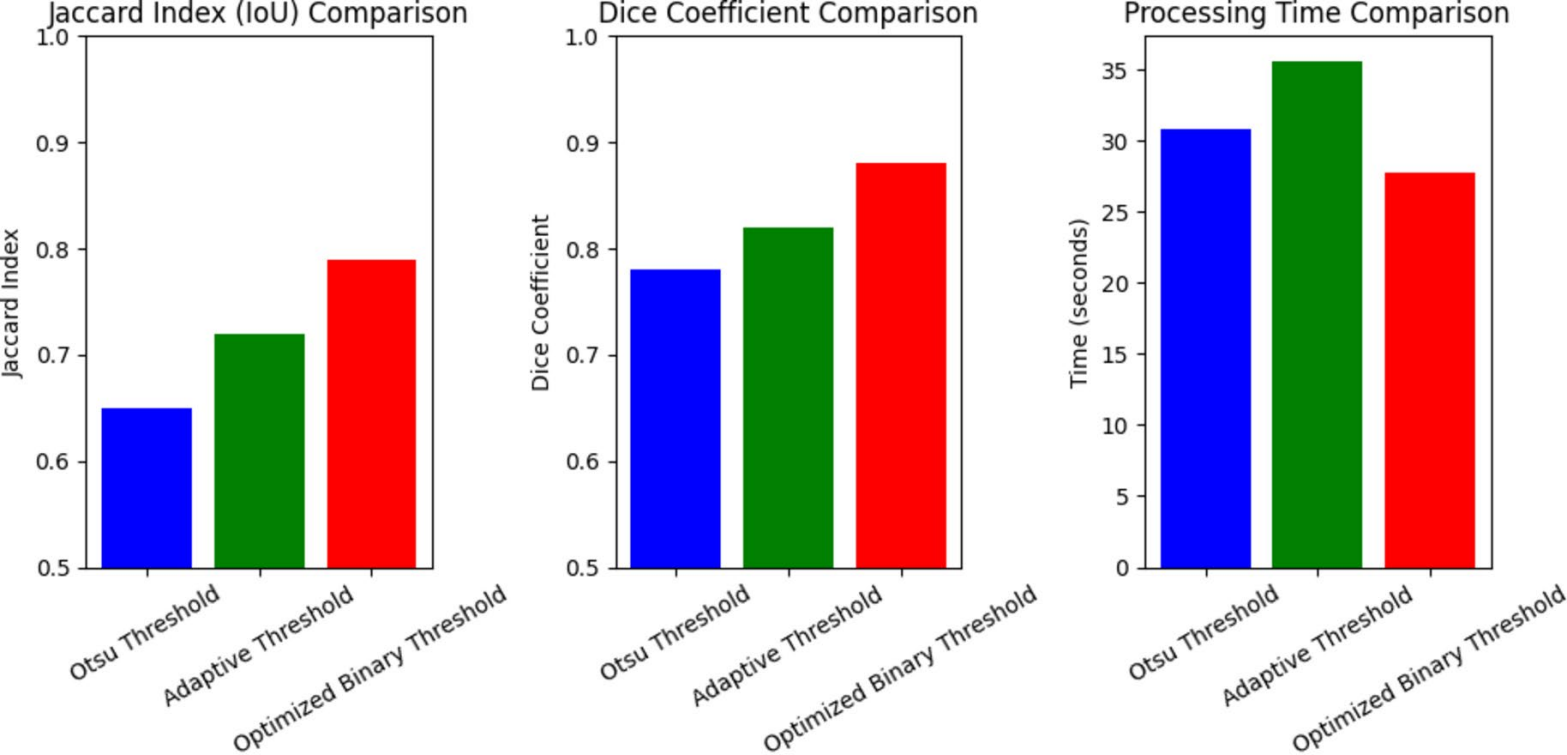
Comparative analysis of the thresholding techniques.

The comparative analysis of the optimal binary thresholding by comparing with existing thresholding techniques shown in Fig. 15. Here, the comparison is done by validating with the Jaccard index, dice coefficient and processing time by utilizing with Otsu’s thresholding, and adaptive thresholding. The advanced RHDAO optimization algorithm is performed by utilizing the optimal threshold value in binary image segmentation. Main objective of the optimization algorithm enables to enhance the segmentation accuracy by comparing with baseline models like Otsus’ thresholding and adaptive thresholding model. Focusing Otsu’s thresholding model, threshold value is automatically selected by maximizing the variance among the foreground and background pixels. By comparing with the jaccard index, the Otsu’s thresholding shows poor performance. Yet, it suffers with non-uniform illumination and complex backgrounds. The adaptive thresholding estimates the diverse threshold values based on local pixel intensity. However, it is computationally expensive in large images. In this research work, the utilization of a hybrid RHDAO algorithm tends to dynamically adjust the threshold values to provide better segmentation performance. Thus, it handles an uneven illumination and minimizes noise than Otsu’s and adaptive models to enhance the speed and processing time, especially in medical imaging tasks. Throughout this analysis, the developed model shows effective performance while compared with baseline thresholding techniques.

### Comparative analysis of the pre-processing techniques

Table 5 elucidates the comparative analysis of pre-processing techniques while comparing with CLAHE and the histogram equalization model. Various performance measures are taken for the validation, in which it proves the effectiveness in the pre-processing phase. By combining the CLAHE + histogram equalization, the accuracy of the model shows 94.90%. Enhancing the accurate performance provides the model more reliable and provides a clean and better pre-processed image. Removing irrelevant background and noises in the thermography image can provide better outcomes. In clinical practice, medical professionals can provide better diagnosis in order to prevent from breast cancer.

**Table 5:** Comparative analysis of pre-processing techniques.

Metrics	CLAHE^41^	Histogram equalization^42^	CLAHE + Histogram equalization
Accuracy	91.67	92.67	94.90
Recall	91.58	92.57	94.94
Specificity	91.75	92.77	94.86
Precision	91.95	92.94	95.00
FPR	8.25	7.23	5.14
FNR	8.42	7.43	5.06
NPV	91.75	92.77	94.86
FDR	8.05	7.06	5.00
F1-Score	91.77	92.75	94.97
MCC	83.33	85.33	89.80

### Ablation study of the developed model

Ablation study analysis of the developed model is compared for diagnosing breast cancer is shown in Table 6. Here, the ablation study is compared and validated utilizing the diverse performance measures to prove the efficiency of the developed model. In this table analysis, the developed RHDAO-StackVDRNet model shows 97.06% in terms of accuracy by neglecting the errors while diagnosing breast cancer. Accurate performance shows the developed model provides better performance while optimizing the parameters to maintain its stability.

**Table 6:** Ablation study of the developed model.

Metrics	StackVDRNet-VGG	StackVDRNet-NoFusion	StackVDRNet-Shallow	StackVDRNet-NoRHDAO	RHDAO-StackVDRNet
Accuracy	89.43	90.40	91.50	93.94	97.06
Recall	89.41	90.47	91.39	93.83	97.00
Specificity	89.45	90.33	91.62	93.97	97.07
Precision	89.71	90.59	91.81	75.67	86.87
FPR	10.55	9.67	8.38	6.03	2.93
FNR	10.59	9.53	8.61	6.17	3.00
NPV	89.45	90.33	91.62	98.70	99.39
FDR	10.29	9.41	8.19	24.33	13.13
F1-Score	89.56	90.53	91.60	83.78	91.65
MCC	78.86	80.80	83.00	80.81	90.07

### Area under curve - receiver operating characteristic (AUC-ROC) curve analysis

**Fig. 16:**
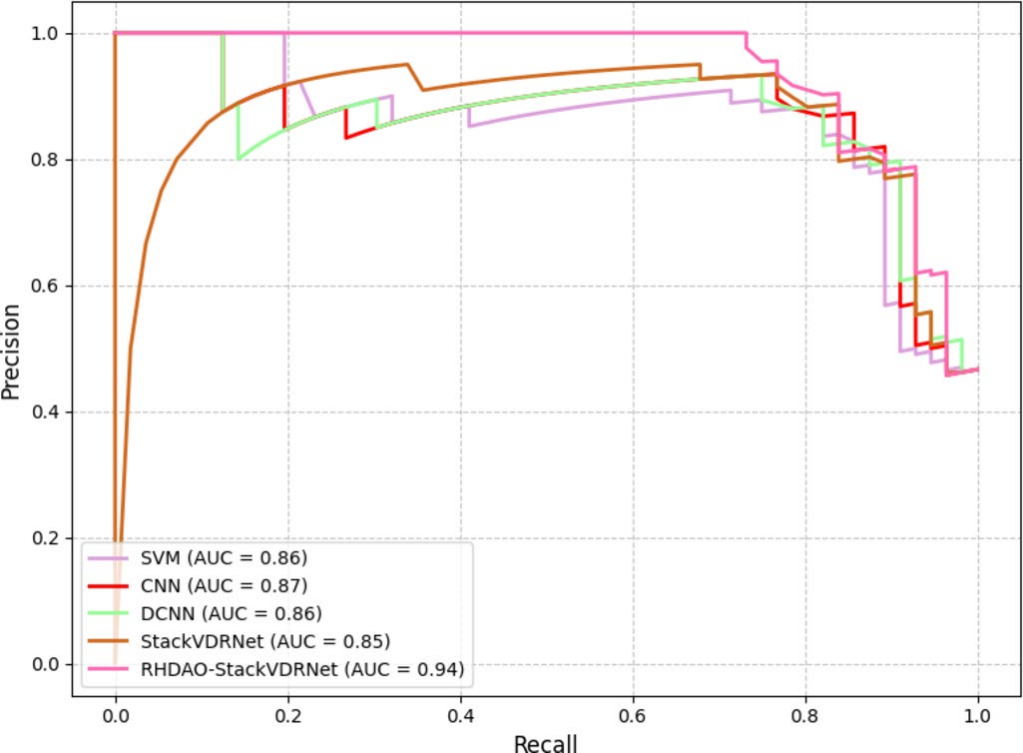
AUC-ROC analysis of the developed model.

Figure 16 depicts the AUC-ROC analysis of the developed model in terms of comparing with precision and recall with diverse classifiers. Here, the analysis of AUC-ROC tends to solve the issue of class imbalance to make the model more effective. With diverse threshold levels, the precision-recall analysis provides more insightful performance when the positive class is limited while comparing with the negative class. The primary concern for this analysis tends to improve the accuracy performance.

### Comparative analysis of the developed model using thermography images

Table 7 shows the comparative analysis of the recommended RHDAO-StackVDRNet model is compared and validated with existing models using the thermography images. In this comparative analysis, the positive as well as the negative measures are validated by improving the diagnosis performance. Comparing with the existing models, the developed model shows greater performance achieving an accuracy rate of 92.27%. The performance enhancement of the model leads to provide better clinical decision-making performance to cure the disease at an earlier stage. Additionally, the error rates in the existing models have significantly increased making the model inaccurate. Increase of false outcomes leads to misclassification. Throughout the simulation findings, the performance of the developed model provides significant and accurate outcomes.

**Table 7:** Comparative analysis of the developed model using thermography images with diverse existing methods.

Metrics	DLF^18^	Mask *R*-CNN^19^	DCNN^24^	Deep-Wavelet Neural Networks^25^	RHDAO-StackVDRNet
Accuracy	88.63	88.80	90.50	90.97	92.27
Recall	88.82	89.09	90.40	90.86	92.24
Specificity	88.44	88.51	90.60	91.08	92.29
Precision	88.76	88.85	90.82	91.28	92.49
FPR	11.56	11.49	9.40	8.92	7.71
FNR	11.18	10.91	9.60	9.14	7.76
NPV	88.44	88.51	90.60	91.08	92.29
FDR	11.24	11.15	9.18	8.72	7.51
F1-Score	88.79	88.97	90.61	91.07	92.36
MCC	77.26	77.60	81.00	81.93	84.53

### Kappa and good detection rate (GDR) analysis of the model

**Fig. 17:**
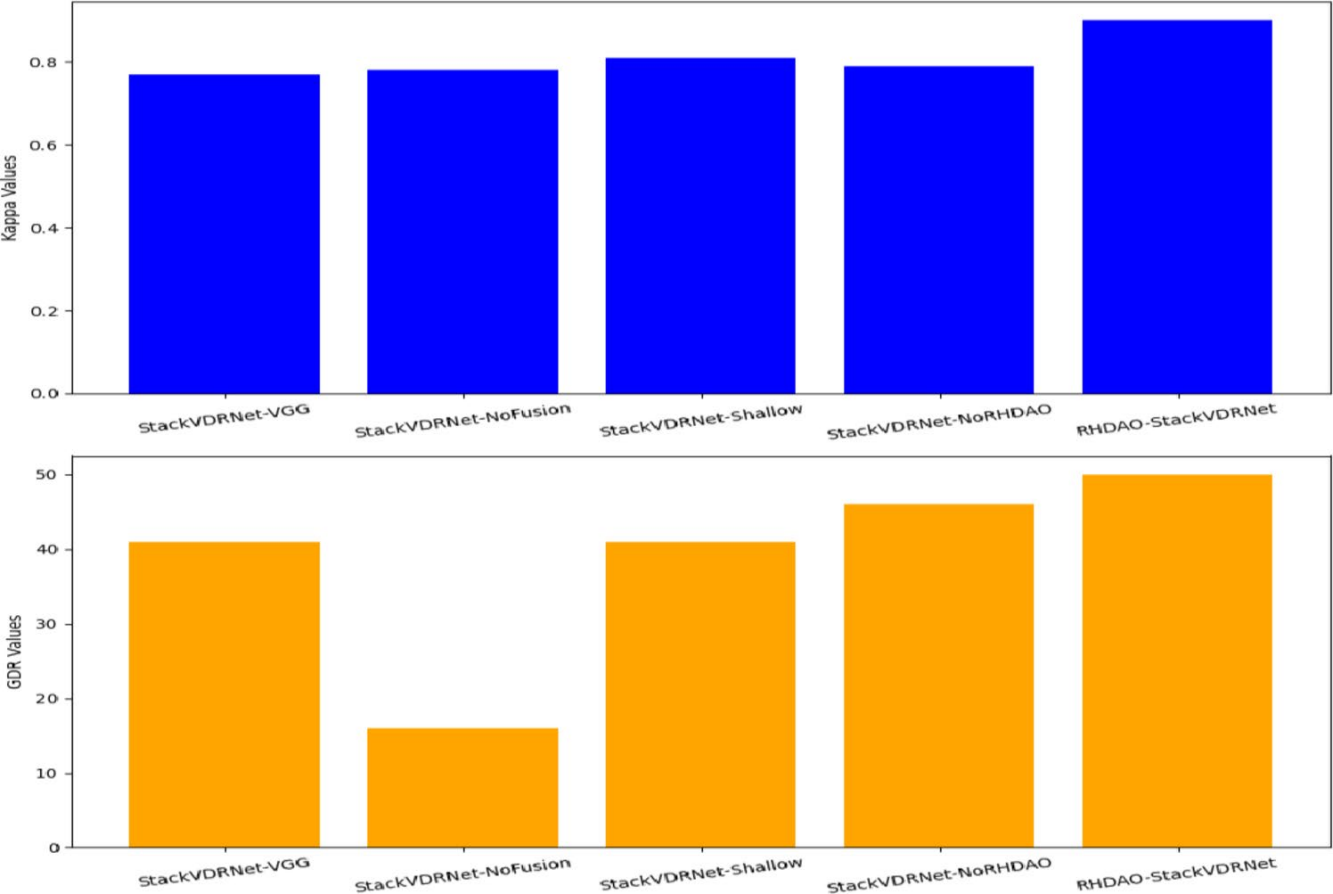
Kappa and GDR analysis of the developed model.

Figure 17 shows the analysis of Kappa and GDR of the developed model while comparing with existing techniques. Kappa is one of the statistical measures in order to evaluate the inter-rater reliability to provide accurate performance. In order to provide better accuracy, the imbalanced issues are taken into the considerations and make better classification outcomes. GDR is the statistical measure to calculate the proportion of correctly identifies positive classes to empower the detection performance. Ensuring a higher GDR value shows better performance for identifying the positive cases.

### Benchmark analysis for training and inference time

For accurately diagnosing breast cancer, the benchmark analysis is validated in terms of training time, inference time, GPU utilization and Memory usage is shown in Table 8. The given table analysis shows developed model provides accurate and reliable outcomes in the clinical settings. Maximizing the training time makes too delay for diagnosing the cancer and fails to provide accurate treatments. On the other hand, utilizing the developed model shows optimal outcomes whereas, it provides faster diagnosis and enhances the patient outcomes by improving the clinical decision-making performance.

**Table 8:** Benchmark analysis of the developed model in terms of training and inference time.

Metrics	Training time (s)	Inference time (ms)	GPU utilization (%)	Memory usage (MB)
Proposed StackVRDNet	950	35	78	1800
DCNN^24^	1200	50	85	2000
ResNet^52^	1400	60	88	2300
DenseNet^53^	1550	65	90	2500
VGG16^51^	1100	55	82	2100

## Conclusion

This paper has explored the adaptive deep learning framework for diagnosing breast cancer using thermography images. Utilizing the thermograph images has the ability to detect the abnormalities been presented in the breast tissue to detect the cancer at an earlier stage. In order to begin the process, the thermograph images were collected from publicly available datasets and then it was given into subsequent pre-processing techniques and segmentation techniques. For effective segmentation, the optimal-thresholding has been utilized for enhancing the performance. From the segmented outcome, the feature extraction has been done with the help of VGG16, ResNet and DenseNet model to get the three set of features. Over the images, the anomalies were detected by using StackVDRNet, in which the hyper-parameters of VGG16, Resnet, and DenseNetwere tuned by the proposed RHDAO. Consequently, the detected images were fed as input to the optimized DA, where the abnormalities were localized effectively. The parameter in DA was also be optimized by RHDAO to effectively locate the abnormal events and improve the diagnosis performance. Finally, the evaluation was analyzed using distinct measures and compared with classical models. The effectiveness of the developed model is analyzed via the conventional breast cancer diagnosis models in terms of various performance measures. For the diagnosis of breast cancer proposed work has achieved more value than 7.24% for SVM, 7.32% for CNN, 4.89% for DCNN, and 3.51% for StackVDRNet, respectively. Overall, the F1-score and MCC of the developed model has attained 91% and 90%, respectively. Although the developed model provides better performance, it shows some pitfalls that need to be considered in the upcoming works. Data augmentation is also suggested in this research work. The developed model needs to be incorporated into mobile technology, in which it provides better quality and sensitivity for providing efficient treatment in remote areas. Additionally, the risk-free screening method using thermography is required in this developed model for enabling the self-screening model at earlier stage without any involvement for radiologists. These limitations will be provoked and rectify in the upcoming works.

## Data Availability

The data underlying this article are available in the Thermal Images for Breast Cancer Diagnosis DMR-IR”, thermal pictures obtained using the dynamic protocol were utilised to document the process of returning the patient’s body the environment with thermal equilibrium. After cooling breasts with an air stream, 20 sequential photographs were acquired at intervals of 15 seconds. The photos can be found on the website “https://visual.ic.uff.br/dmi” in the Database for Research Mastology with Infrared Image - DMR-IR. Additionally, the approach had perfect accuracy with them. “https://www.kaggle.com/datasets/asdeepak/thermal-images-for-breast-cancer-diagnosis-dmrir”.
